# Null models for comparing information decomposition across complex systems

**DOI:** 10.1371/journal.pcbi.1013629

**Published:** 2025-11-05

**Authors:** Alberto Liardi, Fernando E. Rosas, Robin L. Carhart-Harris, George Blackburne, Daniel Bor, Pedro A. M. Mediano

**Affiliations:** 1 Department of Computing, Imperial College London, London, United Kingdom; 2 Cavendish Laboratory, Department of Physics, University of Cambridge, Cambridge, United Kingdom; 3 Center for Complexity Science, Department of Mathematics, Imperial College London, London, United Kingdom; 4 Sussex Centre for Consciousness Science and Sussex AI, University of Sussex, Brighton, United Kingdom; 5 Centre for Psychedelic Research, Department of Brain Sciences, Imperial College London, London, United Kingdom; 6 Centre for Eudaimonia and Human Flourishing, University of Oxford, Oxford, United Kingdom; 7 Department of Neurology, University of California San Francisco, San Francisco, California, United States of America; 8 Department of Experimental Psychology, University College London, London, United Kingdom; 9 Department of Psychology, University of Cambridge, Cambridge, United Kingdom; 10 Department of Psychology, Queen Mary University of London, London, United Kingdom; 11 Division of Psychology and Language Sciences, University College London, London, United Kingdom; Royal Institute of Technology (KTH), SWEDEN

## Abstract

A key feature of information theory is its universality, as it can be applied to study a broad variety of complex systems. However, many information-theoretic measures can vary significantly even across systems with similar properties, making normalisation techniques essential for allowing meaningful comparisons across datasets. Inspired by the framework of Partial Information Decomposition (PID), here we introduce Null Models for Information Theory (NuMIT), a null model-based non-linear normalisation procedure which improves upon standard entropy-based normalisation approaches and overcomes their limitations. We provide practical implementations of the technique for systems with different statistics, and showcase the method on synthetic models and on human neuroimaging data. Our results demonstrate that NuMIT provides a robust and reliable tool to characterise complex systems of interest, allowing cross-dataset comparisons and providing a meaningful significance test for PID analyses.

## Author summary

How do complex systems process information? Perhaps more interestingly, when can we say two systems process information in the same way? Information-theoretic methods have been shown to be promising techniques that can probe the informational architecture of complex systems. Among these, information decomposition frameworks are models that split the information shared between various components into more elemental quantities, allowing for a more intuitive understanding of the system’s properties. In the field of neuroscience, these measures are often used to gauge the differences between conscious states across health and disease. However, comparing these quantities across datasets is non-trivial, and simple normalisation techniques commonly employed have not been formally validated. In this work, we argue that such methods can introduce bias and result in erroneous conclusions, especially when the data under examination is significantly diverse. Our study sheds light on the origins of this issue, as well as its consequences and shortcomings. Moreover, it offers a rigorous procedure that can be employed to standardise these quantities, enabling more robust cross-dataset comparisons.

## Introduction

What are the emergent phenomena of a complex system, and how do we best quantify them? The interconnected and interdependent nature of the components of such systems, as well as their large number of degrees of freedom, are at the same time both their most compelling feature and their greatest challenge. These traits make complex systems unique in their ability to display emergent properties, in which the collective interactions can give rise to novel, unexpected, and self-organised phenomena that cannot be easily predicted by examining the individual parts in isolation. Examples of this abound in ecology [1], economics [2], neuroscience [3], and even in situations from everyday life such as traffic jams [4].

However, this richness in behaviour also poses great challenges to the investigation of their properties. A recent strategy to tackle this problem consists of describing the interactions between a system’s elements by studying how information is routed and processed by each component of the system—an approach known as *information dynamics* [5]. Within this field, a promising approach to unravel the relations among the constituents of complex systems is *Partial Information Decomposition* (PID) [6]. This mathematical framework aims to characterise the interdependencies within a system by breaking down the information that two or more parts provide about another into unique, redundant, and synergistic contributions. A prominent feature of PID, inherited from information theory, consists in its broad applicability—as the decomposition can be calculated on a wide variety of systems. This allows systematic comparisons of the interdependencies exhibited by different systems, and also between different states of the same system. In fact, PID has proven particularly useful to study artificial neural networks [7–11], gene regulatory systems [12,13], cellular automata [14,15], and neural dynamics [16–21].

Despite its attractive features, PID suffers from some shortcomings that potentially limit its applicability. First, despite outlining the relations between the different modes of interdependency, the PID framework does not prescribe a unique functional form to calculate these quantities—which has given rise to numerous proposals of how to best define these measures (see e.g. Refs [6,9,22–30]). Additionally, results obtained through these various approaches can differ, potentially leading to seemingly contradictory conclusions (although, in many practical cases, several measures can yield qualitatively similar results [16,30]). Moreover, it is highly non-trivial to compare PID quantities across datasets, as their values are inherently dependent on the mutual information (MI) of the variables taken into consideration, a quantity that can vary greatly between systems with similar properties, and even more across various datasets. Hence, directly comparing PID atoms belonging to different distributions may yield results purely dictated by the difference in mutual information. To overcome this issue, a normalisation procedure is needed to quantify the amount of synergy, redundancy, and unique information, relative to the MI of the system.

Along this line, this paper introduces a novel methodological approach to allow more sensible PID comparisons of diverse systems’ dynamics, alleviating both shortcomings mentioned above. Drawing from an established approach in network theory [31,32], we introduce a null model technique for information-theoretic estimators that allows the comparison of quantities belonging to different information distributions, while also opening the way to more principled and effective cross-dataset comparisons. We also present a set of theoretical and empirical results that show that our method provides more robust conclusions than standard linear normalisations based on the MI of the system [33–36]. Furthermore, applications to real neural systems show that the proposed technique leads to consistent results across various datasets, even when using different PID formulations.

The rest of this article is structured as follows. We first describe the problem in section *The problem: Comparing PID atoms between systems*: focusing on a practical example, we show the non-linear behaviours of the PID atoms for different MI, discussing the limitations this poses for comparisons across systems. We then provide a solution in section *The solution: A null-model approach to normalise PID atoms* in the form of a null model for normalising PID results. After validating the method on synthetic models (section *Validation on synthetic systems*), we apply it to brain-scanning (magnetoencephalogram; MEG) data of subjects in altered states of consciousness (section *Case study: Information decomposition in cortical dynamics*). Finally, section *Discussion* concludes with a discussion of implications and limitations. Methods are described in detail in section *Materials and methods*.

## The problem: Comparing PID atoms between systems

This section presents the key problems tackled in this paper: the necessity of normalisation techniques for PID comparisons across different systems (section *A simple example*), and addressing the shortcomings of naive normalisation approaches that can lead to misleading results (section *Shortcomings of previous approaches*). We ground our intuitions on simple Gaussian systems, which let us investigate the behaviour of the PID atoms in a tractable manner.

### A simple example

Given a system with two *source* variables X,Y and one *target* variable *T*, PID proposes a decomposition of mutual information into four terms, or *atoms*, as

I(X,Y;T)=Red(X,Y;T)+Un(X;T⧵Y)+Un(Y;T⧵X)+Syn(X,Y;T) . (1)

The information associated to these atoms is commonly described as *redundant* (information provided by both sources separately), *unique* (provided by one source, but not the other), and *synergistic* (provided only by both sources together, but neither of them in isolation). We refer to the quantity I(X,Y;T) as the total mutual information, or TMI.

Unless otherwise specified, for the following analyses we employ the Minimal Mutual Information (MMI) PID [25], in which redundancy reads

Red(X,Y;T)=min(I(X;T),I(Y;T)), (2)

and the rest of the atoms follow from the defining PID equations (c.f. Eqs (17), (18), and (19)). However, our results also apply to other PID measures (Appendix A in S1 Appendix). We refer to section *Materials and methods* for a more in-depth discussion on this topic.

To develop our intuitions, consider two jointly Gaussian univariate sources $S=(X,Y){\;\;}^{T}∼𝒩(0,\Sigma_{S})$ and a one-dimensional target *T* given by

$$T=A\;(\begin{array}{c} X\\ Y \end{array})+\sqrt{g}\varepsilon \;,$$ (3)

where A is a fixed 1×2 matrix of coefficients, also known as connectivity weights, $\varepsilon ∼𝒩(0,\Sigma_{\varepsilon })$ is a white-noise term, and $g\in {\mathbb{R}}^{+}$ is a parameter that determines the level of noise in the system. Intuitively, *A* and $\Sigma_{S}$ describe *how* the sources convey information about the target, while *g* controls *how much* information they provide. If *A* and $\Sigma_{S}$ are fixed, the overall informational structure between *S* and *T* remains untouched—although the TMI changes with different *g*. Therefore, we would expect that as *g* increases the value of all atoms should decrease (as per the data processing inequality)—but at least the overall qualitative character of the system (e.g. whether it is synergy- or redundancy-dominated) should not vary. As an illustration, in the rest of this section we adopt the following values:

$$A=(\begin{array}{cc} 0.5 & 0.5 \end{array})\;\Sigma_{S}=(\begin{array}{cc} 20 & 10\\ 10 & 20 \end{array})\;\Sigma_{\varepsilon }=1\;.$$ (4)

**Fig 1:**
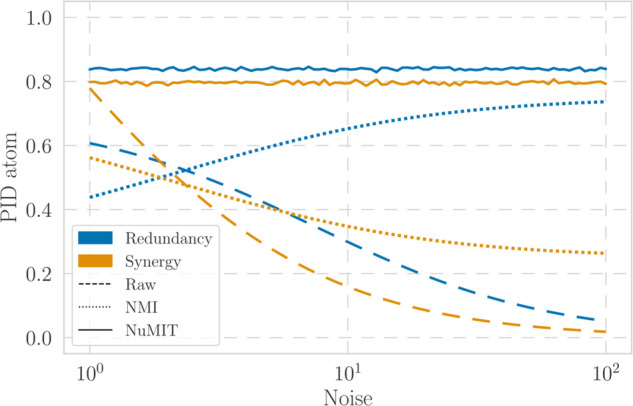
Synergy and redundancy values for the bivariate Gaussian system given by Eqs (3) and (4) for g∈[1,100]. Line styles represent raw atoms (dashed), atoms normalised by total mutual information (NMI, dotted), and atoms normalised by our proposed null-model procedure (NuMIT, solid).

Calculating the MMI-PID for a range of values of *g* shows that this is, surprisingly, not the case (see Fig 1). Although raw values of PID atoms do decrease with *g*, the relative proportion between them changes radically, to the extent that (according to the raw PID values) higher *g* causes the system to switch from being synergy- to redundancy-dominated. This counterintuitive result is an example of a general and pervasive phenomenon, as similar behaviour is observed in higher-dimensional systems and using other PID measures (Appendices C and D in S1 Appendix).

We argue that this behaviour is an important problem for the comparison of PID values across different systems, or across different conditions of the same system. For instance, if one were to observe data from two systems with the same source-target relationship ($A,\Sigma_{S}$) but different levels of signal-to-noise ratio (*g*), one may come to completely opposite conclusions. Therefore, this example illustrates the key problem we tackle in this paper: that raw values of PID atoms are often not comparable across systems with different values of TMI.

### Shortcomings of previous approaches

The naive approach to obtain ‘normalised’ PID atoms that do not depend so strongly on TMI is to simply divide each atom by TMI, i.e.

$${\text{Red}}_{\text{NMI}}(X,Y;T)=\frac{\text{Red}(X,Y;T)}{I(X,Y;T)}\;,$$ (5)

and similarly for all other atoms. We refer to this procedure as *Normalising by Mutual Information* (NMI). NMI makes intuitive sense, is simple, and has the advantage that the resulting values can be understood as the proportion of the TMI that is contributed by each atom.

Unfortunately, however, results from the same Gaussian system as above show that NMI fails to solve the problem we aim to address (dotted lines in Fig 1). NMI atoms still vary widely and show a switch from a synergy- to a redundancy-dominated decomposition as the noise increases. Hence, although this method seems a natural choice for PID normalisation, these results suggest that it might not be appropriate when comparing systems with large differences in TMI.

To see why this happens, note that NMI entails a strong, yet implicit assumption: that the values of PID atoms grow linearly as TMI increases, and therefore that dividing by TMI would yield a value that does not depend on TMI itself. This would seem to make intuitive sense, since the atoms are indeed linearly related to TMI (c.f. Eq (1)). The key issue is that, as we show below, not all atoms grow in the same proportion, contradicting the implicit assumption of NMI.

### On the distribution of PID atoms

If not linearly, how do the different atoms grow as the TMI increases? In this section, we explore the relationship between the atoms and TMI, to show explicitly why NMI fails and to obtain important insights for our solution in section *The solution: A null-model approach to normalise PID atoms*.

We proceed by considering an ensemble of Gaussian systems with different $A,\Sigma_{S}$ that all yield the same TMI. Performing a PID decomposition on each provides a distribution of PID atoms that indicates the most likely values of synergy, redundancy, and unique information for systems with that specific value of TMI. In practice, we do this by randomly sampling each element of the coefficient matrix *A* i.i.d. from a Gaussian distribution, $\Sigma_{S}$ from a Wishart distribution, and finding the value of *g* that results in the desired TMI value. We refer to section *Null model normalisation* for the detailed description of the mathematical procedure followed.

**Fig 2:**
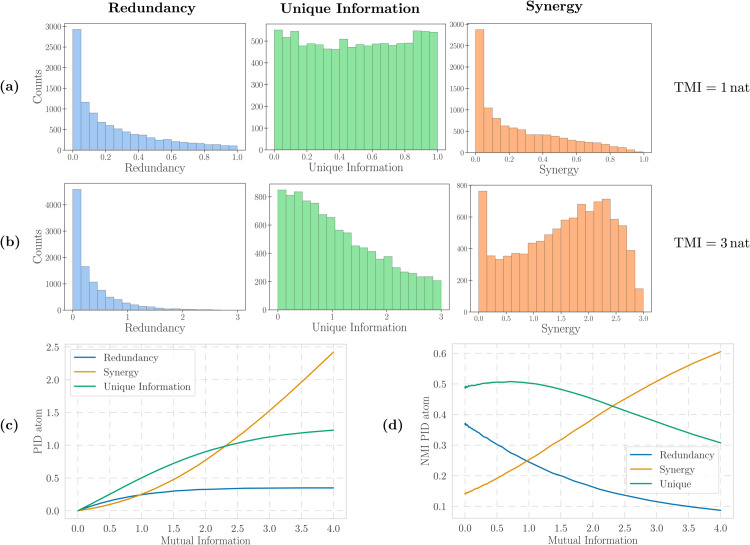
(a) Distributions of redundancy, unique information, and synergy with MMI definition for TMI=1.0 nat and (b) TMI=3.0 nat. (c) PID-atom averages for different values of mutual information over random Gaussian systems with 2 univariate sources and a univariate target. (d) Same as (c) but with NMI-normalised PID atoms.

To develop our intuitions, we begin by inspecting the distribution of each PID atom across Gaussian systems with two particular values of TMI (Fig 2a and 2b), drawing 10,000 samples from the ensemble in each case. From these, we observe that most Gaussian systems with TMI=1 nat are dominated by unique information, and high values of both synergy and redundancy are relatively rare. However, these distributions look very different among systems with TMI=3 nat, which are clearly synergy-dominated, with large values of either redundancy or unique information becoming less likely. This indicates that the PID atoms behave qualitatively differently for different values of TMI, a feature that linear normalisation techniques are unable to capture.

As a further experiment, we repeat this procedure for a range of TMI values between 0 and 4 nat, calculating the mean of the distribution for each atom (Fig 2c). This shows that neither synergy, redundancy, nor unique information grows linearly with TMI. In fact, for systems with low TMI, on average, contributions to mutual information are mainly due to unique information and redundancy, whereas the synergistic component dominates for large TMI. The rapid growth of synergy for TMI above approximately 3 nat suggests that the information in systems with large TMI is mainly composed of synergistic contributions. This can be easily understood in the limit of noiseless systems. If *T* is a deterministic function of X,Y, then TMI is infinite, while the marginal mutual information of both *X* and *Y* remains finite—thus, synergy must be infinite. It is worth noting that increasing the number of sources (i.e. taking X,Y multivariate) exacerbates this phenomenon, as the non-linear behaviour becomes more significant with the dimensionality of the sources (see Fig J in S1 Appendix).

Taken together, the results in this section highlight some problems inherent to the comparison of PID atoms between systems with different TMI, and show why the naive NMI approach is not sufficient. Hence, a more sophisticated normalisation technique is needed.

## Results

### The solution: A null-model approach to normalise PID atoms

#### Null model normalisation.

To address the problems raised in the previous section, we now present the central result of this work: a normalisation procedure that remains unaffected by the amount of noise in the system. In other words, given systems that only differ in the noise level but not in the statistical relationship between source and target variables, the desired method yields qualitatively similar PID decompositions.

Inspired by the use of null models in network science and neuroscience [32,37], the core idea of our method is to compare the PID atoms of the system of interest against those of *an ensemble of all possible systems with the same TMI*. In practice, we can operationalise this idea through the following algorithm:

1. Given the specific system under examination p(S,T), calculate its TMI and perform the PID.
2. Sample a null model *q*_*i*_(*S*,*T*) that has the same TMI as p(S,T) but is otherwise random, and compute its PID.
3. Repeat the previous step *N* times for many sampled *q*_*i*_, obtaining a null distribution of each PID atom.
4. The relative amount of synergistic, unique, or redundant information of *p* can be quantified by taking the quantile of the PID atoms of *p* w.r.t. the null models $\{q_{i}{\}}_{i=1}^{N}$.

We call this approach *Null Models for Information Theory* (NuMIT).

Besides calculating the PID itself, the challenge of the algorithm above is step 2: taking a random sample from the set of probability distributions with a given TMI. We solve such a constraint by introducing a real parameter that, while not changing the underlying statistical structure of the model, can be tuned to yield the desired value of TMI.

#### The Gaussian case.

For simplicity, we show below the mathematical formulation of the method specifically for Gaussian systems. A formulation for autoregressive and discrete models is provided in section *NuMIT normalisation for VAR models* and Appendix F in S1 Appendix, respectively.

Consider a multivariate Gaussian system p(S,T) consisting of two multivariate sources X,Y of dimension *d*_*X*_ and *d*_*Y*_, denoted jointly as $S=[X^{T}\;Y^{T}{]}^{T}$ with dimension $d_{S}=d_{X}+d_{Y}$, such that $S∼𝒩(0,\Sigma_{S})$, and be T a *d*_*T*_-dimensional target variable given by

$$T=AS+\sqrt{g}\varepsilon \;,$$ (6)

where *A* is a $d_{T}\times d_{S}$ coefficient matrix, ε is a multivariate white-noise term $\varepsilon ∼𝒩(0,\Sigma_{\varepsilon })$, and *g* is a non-negative real parameter that controls the noise strength. Following from Eq (6), the covariance matrix of the target reads

$$\Sigma_{T}=A\Sigma_{S}A^{T}+g\Sigma_{\varepsilon }\;.$$ (7)

Therefore, the total mutual information of *p* can be written as

$$\text{TMI}:=I(S;T)=\frac{1}{2}\log\left|\Sigma_{T}\right|-\frac{1}{2}\log\left|g\Sigma_{\varepsilon }\right|\;=\frac{1}{2}\log\left(\frac{\left|A\Sigma_{S}A^{T}+g\Sigma_{\varepsilon }\right|}{\left|g\Sigma_{\varepsilon }\right|}\right)\;.$$ (8)

We can create random systems $q_{i}(S,T)$ by sampling both random coefficients $\tilde{A}$ and random covariance matrices ${\tilde{\Sigma }}_{S},{\tilde{\Sigma }}_{\varepsilon }$. One natural choice is to generate coefficient matrices with elements sampled i.i.d. from a Gaussian distribution ${\tilde{A}}^{ij}∼𝒩(0,1)$, and covariance matrices from Wishart distributions ${\tilde{\Sigma }}_{S}∼W_{d_{S}}({𝟙}_{d_{S}\times d_{S}},d_{S})$, ${\tilde{\Sigma }}_{\varepsilon }∼W_{d_{T}}({𝟙}_{d_{T}\times d_{T}},d_{T})$.

Finally, we can choose the parameter *g* to obtain a null model *q*_*i*_ that has a specific value of total mutual information. We do this through an optimisation procedure, noting that from Eqs (7) and (8) with straightforward calculations we obtain the function *f* whose root determines the value of *g*:

$$f(g)=\left|𝟙+\frac{1}{g}{\tilde{\Sigma }}_{\varepsilon }^{-1}\tilde{A}{\tilde{\Sigma }}_{\text{ S}}{\tilde{A}}^{T}\right|-e^{-2\cdot \text{TMI}}\;.$$ (9)

It is straightforward to show that this is a monotonically decreasing function of the scalar parameter *g*, and therefore can be optimised with off-the-shelf tools. We use the fzero solver in Matlab (R2021b, MathWorks, Natick, MA, USA), although we expect many other solvers to work too. The sampled matrices $\tilde{A},{\tilde{\Sigma }}_{S},{\tilde{\Sigma }}_{\varepsilon }$, together with the resulting value of *g*, determine a null system *q*_*i*_. We can then calculate the PID atoms of *q*_*i*_ and repeat this process multiple times to obtain the null distributions of each PID atom.

#### Validation on synthetic systems.

To illustrate the effectiveness of NuMIT, we investigate how accurately null-normalised PID atoms capture the statistical interdependencies of synthetic systems, also comparing their performance to that of NMI atoms. To achieve this, we consider three simple Gaussian systems for which an intuitive understanding is available, allowing us to directly link the structural features of each model to the expected patterns of information decomposition.

**Fig 3:**
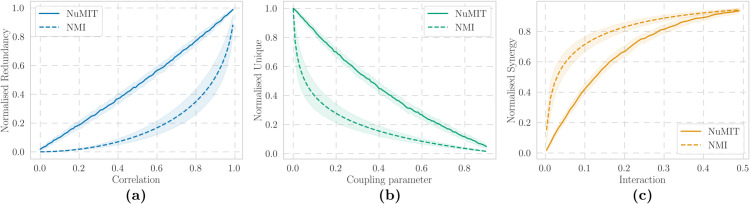
NuMIT- and NMI-normalised PID atoms for Gaussian models of Eq (6) for various choices of parameters. (a) Normalised redundancy as a function of sources correlation (Eq (10)). (b) Normalised unique information and (c) normalised synergy for various cross-coupling values between one source and the target (Eq (11)). Shaded areas represent the standard deviation across various g∈ℝ.

We begin by analysing how normalised redundancy changes when the correlation between the sources varies, expecting the two to be highly correlated. To implement this, we consider

$$A=(\begin{array}{cc} 0.01 & 0.99 \end{array})\;\;\Sigma_{S}=(\begin{array}{cc} 1 & \rho \\ \rho & 1 \end{array})\;\;\Sigma_{\varepsilon }=1\;,$$ (10)

where the large asymmetry in *A* was chosen so that the redundancy of the system is only caused by the correlation between sources. For a given *ρ*, we consider 100 values of $g\in [10^{-2},10^{2}]$ (see Eq (3)), calculate the raw and normalised PID atoms, and compute their average and standard deviation. Repeating the process for various values of ρ∈[0,1], we find that NuMIT-redundancy closely tracks the linear increase in correlation, whereas NMI exhibits a non-linear response—showing a slow gradual rise in redundancy for low *ρ*, followed by a sharp increase beyond ρ≈0.7 (Fig 3a).

To examine unique information, we focus on a system of two uncorrelated sources coupled through the matrix *A*, varying the strength of one connectivity weight across different levels. Specifically, we define

$$A=(\begin{array}{cc} a & 0.49 \end{array})\;\Sigma_{S}=(\begin{array}{cc} 1 & 0\\ 0 & 1 \end{array})\;\Sigma_{\varepsilon }=10^{-4}\;.$$ (11)

In this scenario, we expect source *Y* to provide a large amount of unique information at low values of *a*, with its contribution gradually decreasing and vanishing at *a* = 0.5. Repeating the same procedure as above for a∈[0,0.5], we find that NuMIT-unique information scales faithfully with the coupling parameter. In contrast, NMI exhibits a nonlinear pattern—dropping rapidly and hardly capturing the system’s predominantly unique structure for a≲0.1 (Fig 3b).

Interestingly, the system of Eq (11) can also be employed to test the behaviour of synergy. As unique information decreases for a→0.5, the mutual information of the system is captured by the synergistic component. In particular, synergy is maximised when the coupling parameters are of comparable magnitude, as they strongly entwine the dependencies of *X* and *Y*. As before, we span a∈[0,0.5] and $g\in [10^{-2},10]$, obtaining that in this case as well, the NuMIT-normalised atoms better represent the variability in the autoregressive coefficient, especially for low values of *a* for which NMI-synergy remains non-zero despite minimal coupling (Fig 3c).

Hence, we showed that the NuMIT-normalised PID atoms consistently reflect the underlying structure of the models considered, demonstrating their capacity to capture structural distinctions in the informational architecture of a system. On the other hand, NMI struggles to disentangle the different modes of interdependence, providing a less precise reflection of the system’s organisational features.

### Case study: Information decomposition in cortical dynamics

The basic examples studied above show that the NuMIT-normalised PID atoms correctly quantify the information structure in simple systems. In this section, we analyse real-world brain activity data of subjects during altered states of consciousness to show that our method can reveal new insights about complex systems under study.

#### Motivation and analysis set-up.

Information theory provides effective methods to assess important questions in the field of computational neuroscience, including the assessment of various aspects of cognition and consciousness. For instance, significant advances have been achieved in using complexity measures to characterise altered states induced by psychoactive substances [38–40]. Complementing these studies, here we explore the relationship between brain dynamics and conscious states by decomposing the information structure of such conditions with PID. One particularly interesting case is the change in neural activity elicited by psychedelic drugs like the serotonergic agonists LSD [41] and psilocybin [42] and the NMDA antagonist ketamine [43]. Previous works have reported a decrease in information flow due to these drugs [44], as well as a concomitant decrease in TMI between past and future [45]—prompting the question of whether, or to what extent, the decrease in information flow can be explained by the decrease in TMI.

Addressing a similar question but in the context of PID, we analyse resting-state magnetoencephalography (MEG) recordings of subjects under the effects of different psychedelic drugs—ketamine (KET) (N=19) [43], LSD (N=15) [41], and psilocybin (PSIL) (N=14) [42]—and matching placebo recordings. More details about the datasets and pre-processing pipeline are provided in section *Neural data*, the open data repository [46], and in the original studies [41–43]. Since the data is in the form of a multivariate time series of brain activity, we model it using a Vector Autoregression (VAR) process. VAR models are powerful and versatile tools in the time series modelling literature, and they are particularly attractive for information-theoretic analyses due to their tractability [47–49]. NuMIT normalisation presented in section *Null model normalisation* can be naturally extended to VAR models—for further mathematical details of the framework and normalisation procedure see Secs. *Vector autoregression model* and *NuMIT normalisation for VAR models*.

#### Null-model normalisation reveals higher synergy under psychedelics.

Our analysis starts from the time series of 90 brain regions source-reconstructed from 271 MEG channels according to the AAL atlas [50]. For every subject, drug (ketamine, LSD, or psilocybin), and condition (drug or control), we sample 1000 sets of 10 random regions. Each set is then divided into two groups of 5 regions, and a VAR(1) model is fitted. From this system, we calculate the raw PID atoms as well as those normalised using NMI and NuMIT, using the past states of the 5+5 regions as sources and their joint future state as target. The procedure is thus repeated for each of the 1000 sets. Finally, we average each PID atom across all regions to produce a single value for each subject, drug, condition, and normalisation procedure. More details of this procedure can be found in section *Estimating PID from VAR models*.

**Fig 4:**
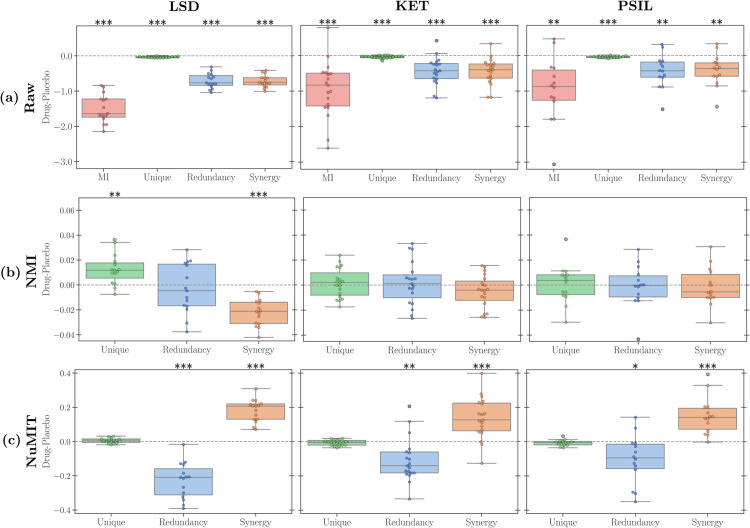
PID-atom distributions for all subjects under different drugs and placebo effects using MMI definition. From left to right, results refer to LSD, ketamine, and psilocybin drugs. Panel rows represent (a) the raw values of PID atoms, (b) the NMI-normalised PID atoms, and (c) the NuMIT-normalised PID atoms. The dashed black lines are drawn at zero. (P-values calculated with a one-sample t-test against the zero-mean null hypothesis. *: *p*<0.05; **: *p*<0.01; ***: *p*<0.001).

The first notable result is that all raw PID atoms decrease in the psychedelic state compared to placebo, consistently across all drugs (Fig 4a). This is expected given the strong (and previously reported [45]) decrease of TMI—since the sum of all atoms decreases between conditions, it makes sense that the atoms that constitute TMI decrease too. Atoms normalised by NMI (Fig 4b) are largely non-significant, with the few significant changes not consistent across drugs. These results call for a suitable normalisation of PID atoms to enable a more meaningful comparison.

Accordingly, we applied our null model normalisation to these findings (Fig 4c). With this technique, we now observe a very consistent pattern of increased synergy and decreased redundancy in the psychedelic state across all three drugs. Therefore, although there is overall less synergy in the psychedelic state, there is in fact more synergy than one would expect given the brain’s TMI.

Although these findings still need further interpretation in the broader context of psychedelic neuroimaging, they show the benefits of our proposed normalisation across diverse datasets. Moreover, analogous results are obtained using different PID definitions, as well as a data-driven approach for generating the null models (Appendix A in S1 Appendix).

#### Null-model normalisation increases consistency across PID measures.

One common concern in experimental applications of PID is the lack of consensus on a one-size-fits-all PID measure. Accordingly, there has been a proliferation of PID measures in the literature (see e.g. Refs [9,23–26] as a non-exhaustive list), raising concerns that different measures may yield conflicting results on the same dataset. Here we argue that our null model normalisation can alleviate this concern, by showing that a proper normalisation makes the results of different PID measures more consistent. We focus here on the comparison between MMI and two other PID methods: Common Change in Surprisal (CCS) [9], and Dependency Constraints (DEP) [26] (Appendix A in S1 Appendix).

As a first analysis, we follow the same procedure as before and compute raw, NMI, and NuMIT-normalised CCS results (Fig B in S1 Appendix). A comparison between CCS and MMI shows that although the distributions of the raw and NMI PID atoms are quite different between the measures, the null-normalised atoms give substantially more consistent patterns. To make this observation more rigorous, we investigate whether CCS and MMI yield consistent conclusions about the effect of psychedelics on brain activity—i.e. whether the difference between drug and placebo conditions on each subject is the same across both PID measures.

To assess whether NuMIT atoms better predict their counterparts across different PID definitions as opposed to NMI, we first implement a Linear Mixed Effect (LME) model of the form

$$\Delta_{MMI}∼Normalisation*\Delta_{CCS}+\;Drug+(1|Subject)\;,$$ (12)

where Normalisation is either NMI or NuMIT, Drug is ketamine, LSD, psilocybin, and $\Delta_{MMI}$ and $\Delta_{CCS}$ denote the difference between atoms in drug and placebo condition from MMI and CCS PID definition, respectively. This LME accounts for variability across subjects by including a random intercept, while also including an interaction term between normalisation and PID definition. Employing the data obtained in the analysis above, we fit the LME of Eq (12) to synergy and redundancy atom separately, obtaining in both cases a statistically significant interaction between normalisation and PID value, with NuMIT consistently yielding higher correlations between CCS and MMI atoms than NMI (see Table 1 under ALL drugs).

**Table 1:** Pearson correlations increase from NMI to NuMIT, for synergy and redundancy and for all drugs (MMI-CCS comparison). P-values refer to the coefficient of the interaction between PID measure and normalisation. ALL refers to the results obtained with the Linear Mixed Effect model (Eq (12)), whereas LSD, KET, PSIL refer to those obtained with the OLS (Eq (29)).

PID atom	Drug	Correlations		p-value
		NMI	NuMIT	
Synergy	ALL	-0.098	0.110	0.001
	LSD	0.071	0.690	0.08
	KET	0.220	0.738	0.08
	PSIL	-0.439	0.790	0.001
Redundancy	ALL	-0.053	0.180	0.001
	LSD	0.488	0.723	0.45
	KET	0.026	0.755	0.02
	PSIL	-0.152	0.760	0.01

**Fig 5:**
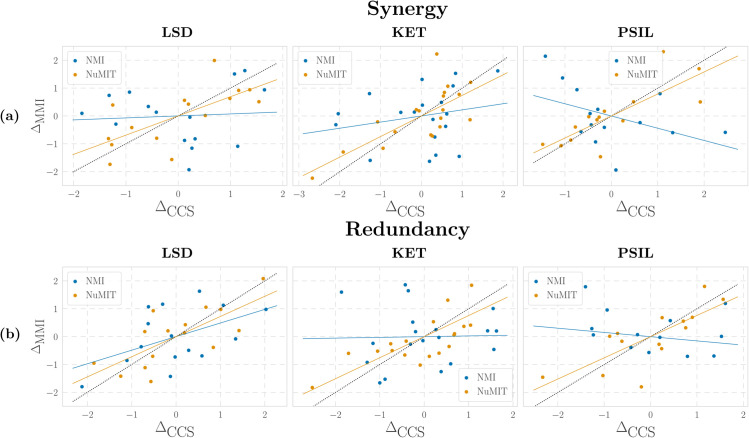
Regression models for NMI- and NuMIT-normalised (a) synergies and (b) redundancies between CCS and MMI PID definitions, for LSD, ketamine, and psilocybin drugs. Δ indicates the differences between drug and placebo in PID atoms obtained with either PID (MMI or CCS).

Having established this overall effect with the mixed model, we then examine each drug separately using an ordinary least squares (OLS) regression. These follow-up analyses are not intended as primary tests, but rather as post-hoc illustrations of the trends uncovered by the LME. Results are shown in Table 1 (under KET, LSD, and PSIL conditions) and Fig 5, confirming that NuMIT normalisation increased the correlations between CCS and MMI definitions across all drugs. Although with this model significance was reached only in some cases, the null model normalisation still increased the correlation between measures to nearly 0.7 and above in all cases. Results with DEP indicate similar trends (Appendix A in S1 Appendix), with slightly smaller correlations with MMI and much higher with CCS (Tables A and B). A complete description of the model and the parameters used is reported in section *Regression model*.

Overall, the LME provides the main statistical evidence that NuMIT improves correspondence between PID definitions, while the per-drug OLS regressions serve as post-hoc confirmation and illustration. Hence, these analyses show that, after suitable normalisation, all three PID measures qualitatively agree on the effects of psychedelics on brain activity, boosting our confidence in the results of the analyses.

## Discussion

### Summary of findings

We first focused on a simple Gaussian model and analysed the effect of the common technique of normalising by mutual information (NMI) each PID atom. Results showed that the distributions of PID components present heavy non-linear dependencies on the total mutual information of the system, contradicting the hidden linearity assumptions behind NMI. As a solution, we suggested a new normalisation method based on the construction of null models. This was performed by generating an ensemble of all systems with the same total mutual information as the system under study, and taking the distribution of PID atoms in this ensemble as the null distribution. Finally, we took the respective quantiles of each atom on its null distribution as universal estimators of how redundant, synergistic, and unique the observed system is. The resulting normalised atoms display greater robustness to noise and generalise more intuitively across systems with different values of TMI.

After outlining the mathematical foundation of the technique, we proved its advantages empirically by applying it to synthetic data and real systems. Direct analyses of neural data showed that NuMIT provided consistent and significant findings, specifically revealing higher synergistic contributions in drug-induced conditions and supporting previous studies in the literature [51]. Such results could not have been obtained with linear normalisation techniques, underlining the importance and necessity of taking into account non-linear PID atoms’ behaviours.

Moreover, a key aspect of an effective normalisation procedure is that it is robust to different ways of calculating PID. In the case of our model, the observations above were validated with multiple PID definitions, showing that the null model approach allows for a more coherent characterisation of the information structure of the system. In fact, a direct comparison between atoms computed with different PIDs showed that the correlations between null-normalised atoms across different PID methods are higher than those obtained with the NMI technique, being consistent for the majority of the methods and drugs.

### Neural null models

The concept of null models has been introduced in network theory [52] as a tool to assess the significance of observed patterns in complex networks. These techniques are designed to generate randomised or controlled versions of a given graph while preserving certain structural characteristics, such as the number of nodes and edges, but introducing randomness in other aspects, such as the arrangement of connections [53]. By comparing network metrics derived from real-world networks to those from null models, it is possible to determine whether the observed features are the result of genuine underlying properties or merely a consequence of random chance. Therefore, null models provide a baseline for statistical inference in network analysis, helping identify patterns and properties such as node degree distributions [54], community structure [55] and detection [56,57], assortativity [58,59], and more.

In this work, we provided a procedure to construct null models for information theory, allowing more rigorous and mathematically robust comparisons and characterisations of information-theoretic quantities. While this technique offers a foundation for better normalisations, it is important to note that the effectiveness of null model comparisons heavily depends on the suitability of the generated null systems to the specific problem under study. In other words, these null models must resemble the original data in some meaningful way, as they could otherwise differ significantly from any real system and be of poor practical use. Therefore, the choice of null models needs to be optimised for each complex system and each experimental hypothesis under consideration.

In our scenario, not knowing a biologically meaningful null model could pose a possible limitation to the study, as real structures of neural null models remain unknown due to the exceptional complexity of the brain. For instance, considering null models significantly different from the real system could prevent a proper characterisation of the feature of interest, which might have been possible if only realistic null systems were taken into account. Nevertheless, if clear and significant results arise, such as the ones presented in section *The solution: A null-model approach to normalise PID atoms*, these are robust indicators of structural information differences.

In practice, for the null normalisation to be effective, the null distributions should closely resemble those of the real system under consideration. The first step is to adopt the same statistical model employed to analyse the original system—e.g. Gaussian or VAR—ensuring that all null distributions lie within the space of distributions the system can generate. Additionally, tuning the parameters of the null procedure could help to choose a suitable null model. This can be achieved by e.g. varying the variance of the Gaussian distribution from which the coefficients *A*^*ij*^ are sampled, using a different family of distributions, or changing the base matrix for the Wishart distribution *W* (section *NuMIT normalisation for VAR models*). However, in our analyses, we found that normalisation results are remarkably robust to the choice of null parameters, suggesting that many different null configurations provide an accurate coverage of the space of possible distributions. This property further supports the universal nature of the NuMIT-normalised quantities obtained. Illustrations and descriptions of these findings are reported in Appendices A and E in S1 Appendix.

### Synergy in Gaussian systems

The concept of synergy has been the subject of interest of various disciplines, giving rise to diverse conceptualisations and methodological approaches. From the early works of Haken [60,61], synergy arises as a property of non-equilibrium systems undergoing phase transitions, where the system exhibits “more than the sum of its parts” through the emergence of order parameters. Following this approach, it is common in the dynamical systems literature to analyse the behaviour of order parameters to assess the presence of synergy, thereby tying the notion to the underlying mechanisms of the system [62–64]. In this sense, synergy is typically regarded as a property of non-linear cooperative dynamics.

In contrast, in information theory, synergy is approached from a statistical perspective. Although grounded in the same intuitive idea of “the whole being more than the sum of the parts” [65,66], this principle is operationalised by analysing the statistical dependencies present in the system’s trajectories, without requiring explicit reference to the system’s dynamical structure. Considering two sources $X_{1},X_{2}$ and a target *Y*, this philosophy can be implemented by studying the difference between the sum of the marginal mutual information and the joint mutual information. Within PID, this reduces to

$$\underset{\text{sum of the parts}}{\underbrace{I(X_{1};Y)+I(X_{2};Y)}}-\underset{\text{whole}}{\underbrace{I(X_{1},X_{2};Y)}}=Red-Syn\;.$$ (13)

The LHS quantity is referred to as co-information or multi-information, and is known to yield negative values in the presence of synergy, in agreement with the PID interpretation. Interestingly, this also happens in linear Gaussian systems [6,92], underscoring that the information-theoretical synergy can also arise in the absence of phase transitions. For this reason, Gaussian models have served as an ideal test bed for exploring PID definitions and behaviours [25,68]. This crucial difference between dynamical and informational synergy highlights the important distinction between studying a system’s structure from a mechanistic versus statistical perspective [67].

Hence, following the information-theoretic interpretation of synergy, in this study we focused primarily on Gaussian systems due to their simplicity and generality, treating them as minimal models that can display non-trivial information dynamics.

### Limitations and future work

A potential shortcoming of the framework proposed here is its inherent dependence on the behaviour of the chosen PID definition across different noise levels. In particular, when the PID measure exhibits non-continuous or non-differentiable behaviour, the resulting normalised quantities can display discontinuities or irregular variations as noise increases (see Fig Gb in S1 Appendix).

Additionally, in this study we specifically focused on VAR(1) models, meaning that we only considered one time step in the past for the analyses of neural dynamics. Future investigations could examine non-Markovian dynamics using VAR(p) models with *p* > 1, which, although already theoretically possible, in practice involves a higher computational load, but might shed light on long-range causal effects within brain activity. Moreover, a fundamental limitation of these models is that they only describe systems with linear dynamics, even though non-linear relationships are essential to understanding and emulating the behaviour of complex systems [69,70]. Thus, further studies should be devoted to exploring more general dynamical processes [71].

Further generalisations of the proposed technique may entail the development of other statistical models, e.g. moving-average or state-space models, and more refined TMI-based information frameworks, like Integrated Information Decomposition (ΦID) [72]. Additionally, the generality of the procedure here performed on TMI enables applications to other core information-theoretic quantities and their decompositions, such as the joint entropy in Partial Entropy Decomposition (PED) [17,73,74] and the KL-divergence in Generalised Information Decomposition (GID) [75].

### Final remarks

We proposed a new methodological framework to quantify the significance of structural measures of information in a complex system. Our approach is based on null models, a method that is widely employed in the analyses of complex networks, but still has not been widely adopted in the context of information-theoretic analyses. This technique has proven useful in understanding non-trivial structures and dynamics, often indicative of meaningful organisation within complex systems.

In this paper, we applied this philosophy to the Partial Information Decomposition framework. The rationale behind this approach is to employ null models to eliminate the intrinsic dependency of information-theoretic quantities on the mutual information of the system, opening the way to comparisons of PID atoms across datasets that show great informational variability.

The results reported in this paper suggest that null-model techniques have great potential for enabling meaningful comparisons of information-theoretic analyses between systems. This is relevant not only for neural systems but also for a wide range of fields, as variations in mutual information can be observed from financial and stock markets [76,77], to ecological systems with different sizes and evolution dynamics [78–80]. Nonetheless, further studies should examine the structure of meaningful null models in these domains and their applications to various real-world datasets.

Overall, we hope the presented findings may foster further investigations on the potential of null models for complementing information-theoretic methods. To encourage the usage of these methods in the information theory community, we provide the code for the null model normalisation in a publicly available GitHub repository: https://github.com/alberto-liardi/NuMIT.

## Materials and methods

### Ethics statement

All data analysed in this work were taken from the open-source repository https://doi.org/10.7910/DVN/9Q1SKM. LSD data was collected in [41], approved by the National Research Ethics Service committee London-West London and was conducted in accordance with the revised declaration of Helsinki (2000), the International Committee on Harmonization Good Clinical Practice guidelines, and National Health Service Research Governance Framework. Ketamine and psilocybin data were presented in [42,43], approved by a UK National Health Service research ethics committee, and conducted with informed consent of the participants. We refer to the original studies for further details.

### Partial information decomposition

Information theory is largely based upon the notion of Shannon entropy, which quantifies the average information content in a random variable [81]. In other words, the entropy of the stochastic variable *X* accounts for the information we gain (on average) about the system after *X* is measured, and it’s defined as

$$H(X)=-\sum_{x}p(x)\log p(x)\;,$$ (14)

where *x* indicates the possible outcomes of *X*. However, entropy alone does not capture the relations between variables in a system. In contrast, mutual information (MI) is a measure of the average information shared between two variables X,Y, and can be interpreted as the reduction in uncertainty about *X* given the knowledge of *Y*. Its definition reads

I(X;Y)=H(X)−H(X|Y), (15)

where H(X|Y) is the entropy of *X* conditioned over *Y*

$$H(X|Y)=-\sum_{y}p(y)\;H(X|y)\;=-\sum_{x,y}p(x,y)\log p(x|y)\;.$$ (16)

However, in a complex system constituted of many elements, mutual information cannot capture high-order interactions as it only describes pairwise relations. Considering a 3-variable system, with sources X,Y and a target *T*, the framework of PID solves this limitation by decomposing pairwise MI into three kinds of quantities: unique information (Un), redundancy (Red), and synergy (Syn):

I(X;T)=Un(X;T⧵Y)+Red(X,Y;T) (17)

I(Y;T)=Un(Y;T⧵X)+Red(X,Y;T) (18)

I(X,Y;T)= Red(X,Y;T)+Un(X;T⧵Y) +Un(Y;T⧵X)+Syn(X,Y;T) , (19)

where I(X,Y;T) is the joint mutual information that X,Y provide about *T*, whereas I(X;T), I(Y;T) are the marginal mutual information of *X* and *Y* respectively.

However, the PID equations do not provide a unique solution to calculate such quantities, as they form an underdetermined system of three equations and four unknowns (Eqs (17), (18), (19)), and various studies have been devolved in finding a suitable expression [6,9,22–30]. In this work, we mainly focused on the *Minimum Mutual Information* (MMI) definition proposed by Barrett [25], who proved that, for 3-variable Gaussian systems, seemingly different versions of redundancy previously suggested in the literature reduce to the simpler and more intuitive expression:

$$\text{Red}(X,Y;T)={\text{Red}}_{\text{MMI}}(X,Y;T)\;=\text{min}(I(X;T),I(Y;T)).$$ (20)

Although this is the central definition used in the work, for the analyses on real data we also considered the *Unique Information via Dependency Constraints* (DEP) by James [26] and the *Common Change in Surprisal* (CCS) definition by Ince [9], showing how our method yields consistent results with all three definitions.

Although in theory the PID decomposition can be applied to a system of *N* sources, in practice this leads to a super-exponential growth of PID atoms and intractable computational loads for high values of *N*. Therefore, throughout this work, we considered an arbitrary number of sources and partitioned them into two multivariate variables X and Y, then employed the 3-variable PID described above.

Hence, studying a system’s information dynamics with PID entails computing the entropies of the variables in the system, then the mutual information between sources and target, and finally proceeding with the PID decomposition.

### Vector autoregression model

Vector AutoRegression (VAR) is a statistical model employed to study multivariate time series. These models allow for tractable analyses of the interdependencies between variables in a complex system [82], and have found broad applications in economics [83], statistics [84], and social sciences [85]. VAR models have already been applied to the study of biological systems [47,48], offering more robust and easier estimations of the covariance of the system compared to direct calculations from the raw signal. In this work, we employed this framework to interpret multivariate time series coming from MEG data, employing VAR to capture the statistical relations between different regions of the brain (section *The solution: A null-model approach to normalise PID atoms*).

The framework consists of a set of linear equations in which the evolution of the system follows a deterministic growth of the past states, plus a noise term. Considering an *n*-dimensional time series ${𝐗}_{t}$, the VAR equation can be written as:

$${𝐗}_{t}=\sum_{l=1}^{p}{\text{A}}_{l}{𝐗}_{t-l}+\eta_{t}\;,$$ (21)

where we introduced the shorthand matrix notation


$${𝐗}_{i}=(\begin{array}{c} X_{i}^{1}\\ \vdots \\ X_{i}^{n} \end{array}),\;\;\;\eta_{i}=(\begin{array}{c} \eta_{i}^{1}\\ \vdots \\ \;\eta_{i}^{n} \end{array}),$$

where *p* is a positive integer, η a multivariate white noise term, and *A*_*l*_ the n×n evolution matrix that contains the *l*-th lag VAR coefficients. In neuroscience, these are also called *effective connectivity matrices*, as they describe the effective interdependencies between different regions of the brain. Notice how the model only takes into account the last *p* steps of the time series, ignoring further past states. For this reason, *p* is named the *model order* and the system is called VAR(p).

Conducting an information dynamics analysis involves studying the covariances between the time-lagged variables. These appear as elements of the autocovariance matrices $\Gamma_{k}\equiv 𝔼[{𝐗}_{t}\;{𝐗}_{t-k}^{T}]$, which can be computed through the Yule-Walker equations [86,87]:

$$\Gamma_{k}=\sum_{l=1}^{p}{\text{A}}_{l}\Gamma_{k-l}+\delta_{k0}V,\;k=0,...,p\;,$$ (22)

where $\delta_{k0}$ is the Kronecker delta, and V the residual covariance, i.e. the covariance matrix of the white noise distribution. Thus, Eq (22) provides a recipe to obtain $\Gamma_{k}$ matrices through a recursive relation. However, instead of solving Eq (22) directly, we can introduce the following quantities

$${𝐒}_{t}=(\begin{array}{c} X_{t}\\ X_{t-1}\\ \vdots \\ X_{t-p+1} \end{array}),\;\;\varepsilon_{t}=(\begin{array}{c} \eta_{t}\\ 0\\ \vdots \\ 0 \end{array}),$$ (23)

$$𝐀=(\begin{array}{ccccc} {\text{A}}_{1} & {\text{A}}_{2} & \cdots & {\text{A}}_{p-1} & {\text{A}}_{p}\\ 𝟙 & 0 & \cdots & 0 & 0\\ 0 & 𝟙 & \cdots & 0 & 0\\ \vdots & \vdots & \ddots & \vdots & \vdots \\ 0 & 0 & \cdots & 𝟙 & 0 \end{array}),$$ (24)

$$\Gamma =(\begin{array}{cccc} \Gamma_{0} & \Gamma_{1} & \cdots & \Gamma_{p-1}\\ \Gamma_{1}^{T} & \Gamma_{0} & \cdots & \Gamma_{p-2}\\ \vdots & \vdots & \ddots & \vdots \\ \Gamma_{p-1}^{T} & \Gamma_{p-2}^{T} & \cdots & \Gamma_{0} \end{array}),$$ (25)

and formally rewrite Eq (21) as a VAR(1) model:

$${𝐒}_{t}=𝐀{𝐒}_{t-1}+\varepsilon_{t}.$$ (26)

From here it follows that Γ needs to satisfy the discrete Lyapunov equation [88] given by

$$\Gamma =𝐀\Gamma {𝐀}^{T}+\text{ W},$$ (27)

where **W** is a block matrix with residual covariance V in the first entry and zeros elsewhere.

Hence, once *A*_*k*_ and *V* are known, it is possible to obtain the autocovariance matrices from Eq (27) and then compute the full covariance matrix of the system.

### Estimating PID from VAR models

In this work, we studied brain activity employing the PID framework and VAR models. Here we provide the technical details of the procedure.

For each subject in each specific condition, the analysis proceeds from sets of 10 brain regions randomly chosen from the dataset, considering their time series across 50 random epochs. The choice of analysing 10 regions out of the available 90 reflects a compromise between maintaining sufficient statistical power and ensuring computational tractability. Moreover, these epochs are treated as independent samples from a Gaussian distribution under the assumption of stationarity, and therefore need not follow temporal order. We then further randomly split the 10 brain regions into two sets of 5 each, and consider their past states as sources X,Y and their joint future state as target *T*. Employing the MVGC2 toolbox [89], we fitted a VAR(1) model to these time series using a Locally Weighted Regression (LWR) estimator, obtaining a 10×10 matrix of the coefficients *A* and a 10×10 residual covariance *V*. Autocorrelation matrices $\Gamma_{k}$ can then be computed through a Lyapunov equation (Eq (27)), and from here the full covariance of the system can be reconstructed. Interpreting the target of the decomposition *T* as the joint future state of the VAR model, and the sources X,Y as the past information of the system, we can calculate the total mutual information (TMI) as

$$I(X,Y;T)=\frac{1}{2}\log\left|\Gamma_{0}\right|-\frac{1}{2}\log\left|V\right|\;,$$ (28)

and proceed with the PID decomposition and any desired normalisation. By repeating the process 1000 times for each subject and condition, we obtain a set of (possibly normalised) PID atoms that quantify the information flow between regions of the brain over time.

**Fig 6:**
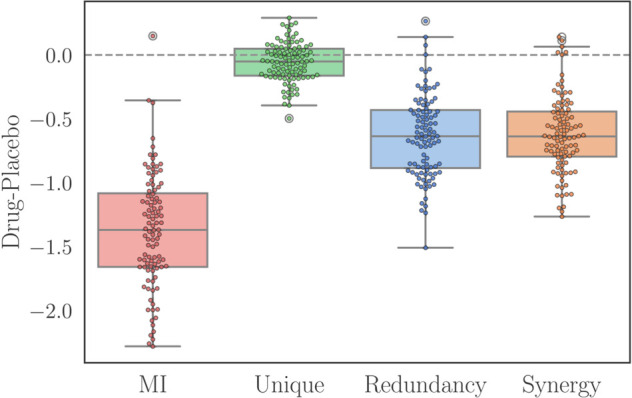
Raw values of PID atoms distribution for subject 1 under ketamine and placebo effects using the MMI PID.

As an example, in Fig 6 we present the resulting distribution of the PID components for the subject S1 under ketamine and placebo, focusing on the difference between atoms obtained in the drug and placebo conditions. It can be already noted that TMI is consistently higher in the placebo condition, thus potentially affecting the overall values of all PID atoms and indicating the necessity of a robust normalisation technique.

Eventually, averaging over these points and repeating the same process for each subject gives the results presented in Figs 4, A, and B in S1 Appendix.

### NuMIT normalisation for VAR models

Here we develop the null model normalisation technique for the VAR model, which was employed for the neural analyses in section *Case study: Information decomposition in cortical dynamics*.

From the defining evolution equation of the model (Eq (21)), we note that the VAR model arises as a non-Markovian generalisation of the Gaussian system studied in section *On the distribution of PID atoms*, in the case in which the target variable is the future state of the sources ($d_{S}=d_{T}$). Therefore, in principle the parameters of the null systems could be sampled analogously to the Gaussian case (section *The Gaussian case*). However, due to the scaling properties of the Lyapunov equation (Eq (27)), rescaling the noise covariance *V* by a factor *g* also affects the covariance matrices in the same way, thus leading to the same TMI. This property of the VAR model intrinsically satisfies one of our requirements for a normalisation procedure, as it avoids the problem of the PID atoms being dependent on the noise of the system. However, it is still necessary to embed a parameter in the model so that PID also becomes independent of the value of the system’s mutual information. To achieve this, we repurpose *g* as the spectral radius of the evolution matrices *A*_*k*_, so that the autocovariances $\Gamma_{k},\;k=0,\ldots ,p$ become implicit functions of *g*. More specifically, given the companion matrix **A** of Eq (24), it is always possible to change its spectral radius to a new value $\rho^{\prime }$ by exponentially decaying its coefficients by a factor of $f=\rho^{\prime }/\rho$. Importantly, such a spectral radius determines the signal-to-noise ratio of the time series, therefore modulating the mutual information exchanged between sources and target. Hence, if we identify *ρ* with the optimisation parameter *g*, we can tune *g* so that the VAR system defined by A,V,p provides the desired TMI. For a VAR(1) model, this procedure simply reduces to rescaling the coefficients of *A* by *f*.

Thus, in practice, once the TMI of the real system p(S,T) is known, for each null model *q*_*i*_(*S*,*T*) we sample $A_{k}^{ij}∼𝒩(0,1)$ and $V∼W_{d_{S}}({𝟙}_{d_{S}\times d_{S}},d_{S})$, then optimise *g* through Eqs (27)–(28) to satisfy the constraint on the TMI. Eventually, marginal mutual information can be calculated and the PID performed.

### Regression model

In section *Null-model normalisation increases consistency across PID measures* we employed a regression model to assess the similarity between different PID definitions depending on the normalisation used. The goal was to observe how accurately a normalised PID atom computed with CCS can predict the corresponding normalised value computed with MMI, both with NMI and NuMIT. This was achieved by building a regression model with two predictors, the CCS PID atoms $\Delta_{\text{CCS}}$—obtained for each subject and normalised with either method—and a binary dummy variable *m*, set to 0 for the NMI normalisation and to 1 for NuMIT. To study the interplay between Δ and m, an interaction term is included. Note that $\Delta_{\text{CCS}}$ corresponds to the difference in the corresponding atom between drug and placebo conditions for a given subject, averaged across all sets of brain regions (similarly for MMI). Mathematically,

$$\Delta_{\text{MMI}}=\beta_{0}+\beta_{1}\;\Delta_{\text{CCS}}+\beta_{2}\;m+\beta_{3}\;\Delta_{\text{CCS}}\;m\;,$$ (29)

where $\beta_{i},\;i=0,1,2,3$ are the regression coefficients. Considering the PID atoms for each subject obtained in Fig 4 and Fig A and B in S1 Appendix, we first standardise the points to mean 0 and variance 1, and then estimate the model parameters $\beta_{i},\;i=0,1,2,3$ along with their p-values. The $\beta_{3}$ term is of particular interest, as it quantifies the extent to which NuMIT normalisation (*m* = 1) increases the correlation between $\Delta_{\text{MMI}}$ and $\Delta_{\text{CCS}}$. The fitted models are shown in Fig 5 and the p-values for $\beta_{3}$ in Table 1.

We performed analogous analyses for the DEP-MMI and CCS-DEP comparisons reported in Appendix A in S1 Appendix.

### Neural data

The data employed in the analysis consist of pharmaco-MEG recordings of patients under LSD [41] (15 subjects), psilocybin (PSI) [42] (14 subjects), and ketamine (KET) [43] (19 subjects) drugs in resting states. Data were obtained from an open data repository [46]. We provide a short overview of the datasets here—interested readers are referred to the original studies for an exhaustive description of the methods and the acquisition details of each experiment.

#### Participants and drug infusion.

All participants gave informed consent to take part in the studies, approved by the UK National Health Service, and were excluded if they were younger than 21 years old, pregnant, had a personal or immediate family history of psychiatric disorder, suffered from substance dependence, or were smokers (only for KET). Moreover, subjects were exempted if they suffered from a medical condition that would render the volunteer unsuitable, such as psychiatric disorders, cardiovascular diseases, claustrophobia, blood or needle phobia, problematic alcohol abuse, and others. Also, patients undergoing LSD and PSIL delivery must have had previous experience with hallucinogenic drugs, but not within 6 weeks of the study.

Drug delivery comprised of intravenous administration of a fixed single dose for LSD and PSIL, and a continued infusion of 40 minutes for KET. PSIL and KET data were obtained immediately after drug delivery, whereas for LSD the data were obtained after 4 hours, due to the slow pharmacodynamics of the drug. Placebo conditions involved an injection of saline solution and were conducted with identical procedures and under identical conditions to the corresponding drug.

#### Data acquisition and preprocessing.

The data were recorded with a 271-gradiometer CTF MEG scan at the Cardiff University Brain Research Imaging Centre (CUBRIC). Each patient underwent two scanning sessions, both in eyes-closed resting state post-administration of drugs and placebo. Source-reconstruction of the data was performed on the centroid of the Automated Anatomical Labelling (AAL) brain atlas [50] using a linearly constrained minimum variance beamformer [90]. Raw data was collected with a sampling frequency of 600Hz and split into 2-second epochs (i.e. 1200 timepoints). Preprocessing was performed with the FieldTrip toolbox [91], and consisted of the rejection of bad epochs, bad channels, and bad ICA components by visual inspection, a lowpass filter at 100Hz and a downsampling to 200Hz. Line noise was removed by fitting a sinusoidal signal at 50Hz and harmonics using a least-squares method, then subtracting it from the data.

#### Data-driven NuMIT normalisation.

With the aim of generating null models which are more faithful to the original system, and obviating the potential shortcomings discussed in section *Neural null models*, here we propose an alternative, data-driven approach to generate the null space based on realistic models. Referring to the Supplementary Material for the application of the technique (Appendix A in S1 Appendix), here we outline the methodological details.

As described in section *NuMIT normalisation for VAR models*, a possible way to construct the null models is to randomly sample the coefficient matrix *A* such that $A^{ij}∼𝒩(0,1)$, the residual covariance $V∼W_{d_{S}}({𝟙}_{d_{S}\times d_{S}},d_{S})$, and then rescale *A* by a suitable $g^{⋆}$ such that null system conveys the desired TMI. However, these null models might deviate substantially from the original system $\left(\tilde{A},\tilde{V}\right)$, leading the atoms of the true system to lie on the edges of the null distribution, thereby limiting the usefulness of the null-based normalisation.

To address this, we propose fixing the coefficient matrix to its empirical estimate, $A^{ij}={\tilde{A}}^{ij}$, rather than sampling it randomly. Then, analogously as before, this matrix is subsequently rescaled by the parameter *g* to reproduce the original TMI. Because the residual covariance *V* is still sampled from a Wishart distribution, and the rescaling *g* affects *A* differently across draws, this strategy ensures broad variability in the null models while ensuring that the null space remains anchored to the real system.

In practice, following the setup outlined in section, once all the 1000 VAR(1) models are computed for all sets of 10 brain regions for a specific subject and drug, this provides us with 1000 10×10 matrices ${\tilde{A}}_{i,DRUG}$ and 1000 10×10 matrices ${\tilde{A}}_{i,PLA}$, which refer to the drug and placebo conditions, respectively. Thus, during the construction of each null model, we randomly select one of these empirical matrices to serve as the coefficient matrix of the null model, which is subsequently rescaled by *g*. Importantly, null models for a given (subject, drug) pair are constructed exclusively from that pair’s empirical coefficients, ensuring consistency and avoiding mixtures across subjects or drugs.

As demonstrated in Appendix A in S1 Appendix, this procedure yields effective null models that both mitigate the issues described above and increase the flexibility of the proposed framework.

## Supporting information

S1 Appendix **Appendix A** contains additional results on neural MEG data obtained with CCS and DEP PID measures and an alternative null construction procedure. **Appendix B** provides further validation of NuMIT on synthetic systems. **Appendix C** includes additional noise sweep analyses for both Gaussian and VAR systems. **Appendix D** presents the behaviour of PID atoms in higher-dimensional systems. **Appendix E** contains further insights into the construction of the null models with various choices of parameters and distributions. **Appendix F** provides a possible implementation of NuMIT for discrete systems.
(PDF)
