# Supplementary material for: Null models for comparing information decomposition across complex systems
Source: PLoS Comput Biol. 2025 Nov 5;21(11):e1013629. doi: 10.1371/journal.pcbi.1013629 (PMC12614810; doi:10.1371/journal.pcbi.1013629)
Supplement: S1 Appendix — Appendix A contains additional results on neural MEG data obtained with CCS and DEP PID measures and an alternative null construction procedure. Appendix B provides further validation of NuMIT on synthetic systems. Appendix C includes additional noise sweep analyses for both Gaussian and VAR systems. Appendix D presents the behaviour of PID atoms in higher-dimensional systems. Appendix E contains further insights into the construction of the null models with various choices of parameters and distributions. Appendix F provides a possible implementation of NuMIT for discrete systems. (PDF) [file pcbi.1013629.s001.pdf]

Supporting Information
S1 Appendix

Null models for comparing information decomposition across complex systems

Alberto Liardi, Fernando E. Rosas, Robin L. Carhart-Harris, George Blackburne, Daniel Bor, Pedro A.M. Mediano

A Remaining MEG results

In this section, we present the results of the analyses of psychedelic MEG data obtained with the DEP and CCS PID formulations.

PID via dependency constraints (DEP)

Unique Information via Dependency Constrains (DEP) [1] is a PID formulation in which the unique information is quantified by a statistical dependency decomposition, i.e. a procedure that assesses how the dependencies between the sources affect the mutual information between sources and targets. In Fig A we present the results of the MEG analysis obtained by using the DEP definition of unique information.

As expected, the higher TDMI found in the placebo condition leads to a decrease in the raw values of all PID atoms under the effects of every drug. While NMI yields results inconsistent across drugs, the results obtained by NuMIT normalisation show consistently higher synergistic values across all three psychedelics.

Finally, note that although raw (Fig Aa) and NMI-normalised (Fig Ab) PID values show contrasting behaviour with the MMI results presented in the main text (Sec. *Case study: Information decomposition in cortical dynamics*), the outcomes of the null model procedure are broadly consistent between different PID measures.

PID via common change in surprisal (CCS)

In Redundant Information with Pointwise Change in Surprisal (CCS) PID, redundancy is quantified by specific local co-information terms drawn from a maximum entropy distribution, providing an intuitive interpretation while also satisfying the core axioms of a redundancy metric, finding broad applications in various areas [2].

Using this formulation, we obtain the results shown in Fig B.

As before, the PID atom distributions calculated with the null normalisation technique appear to be strongly indicative of higher synergistic contributions in the information flow of brains under all three psychoactive substances.

Empirical validation with data-driven null models

As a further proof of how NuMIT can provide robust and significant results across a wide range of systems and null models (see also App. *NuMIT robustness to null parameters* below), we reproduce the empirical analysis on psychedelic neural data by using data-driven null models, restricting to MMI PID for convenience.

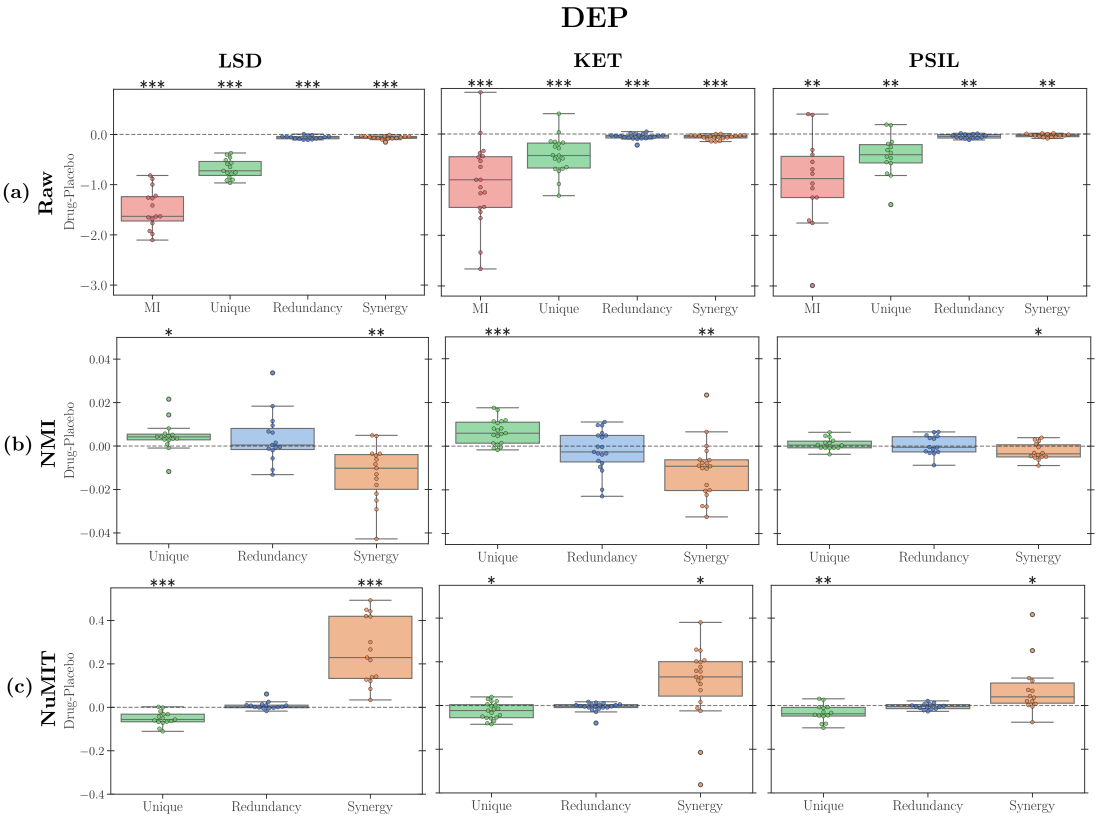

Fig A. PID atoms distribution for all subjects under different drugs and placebo effects using the DEP PID. From left to right, results refer to LSD, ketamine, and psilocybin drugs. Panel rows represent (a) the raw values of PID atoms, (b) the NMI-normalised PID atoms, and (c) the NuMIT-normalised PID atoms. The dashed black lines are drawn at zero. (P-values calculated with a one-sample t-test against the zero-mean null hypothesis. *: $p < 0.05$; **: $p < 0.01$; ***: $p < 0.001$).

As discussed in Sec. *Discussion*, in some scenarios, completely random null distributions might not be appropriate to identify differences in the systems under consideration, due to too large or too small variability of the data, or to a poor choice of the null models constructed. In these cases, however, empirical data-driven approaches for the generation of the null distributions can still be employed. Here we propose a possible approach.

Instead of sampling random coefficient matrices A from Gaussian distributions (Sec. *NuMIT normalisation for VAR models*), we can use the empirical coefficients which were obtained by fitting the VAR models to the real data for the calculation of the raw PID atoms. Hence, for each subject and every construction of a null model, we randomly select an A matrix from that subject's set of empirical coefficients, both from drug and placebo conditions, and then rescale its spectral radius to match the desired TMI (Sec. *NuMIT normalisation for VAR models*). Thus, by grounding the null models in empirical dynamics, this approach can help in the differentiation of the two conditions as it avoids generating null models that are overly dissimilar from the real data. Notably, this method yields results consistent with those shown in Sec. *Case study: Information decomposition in cortical dynamics* (Fig C). A more detailed description of the procedure followed is outlined in Sec. *Data-driven NuMIT normalisation*.

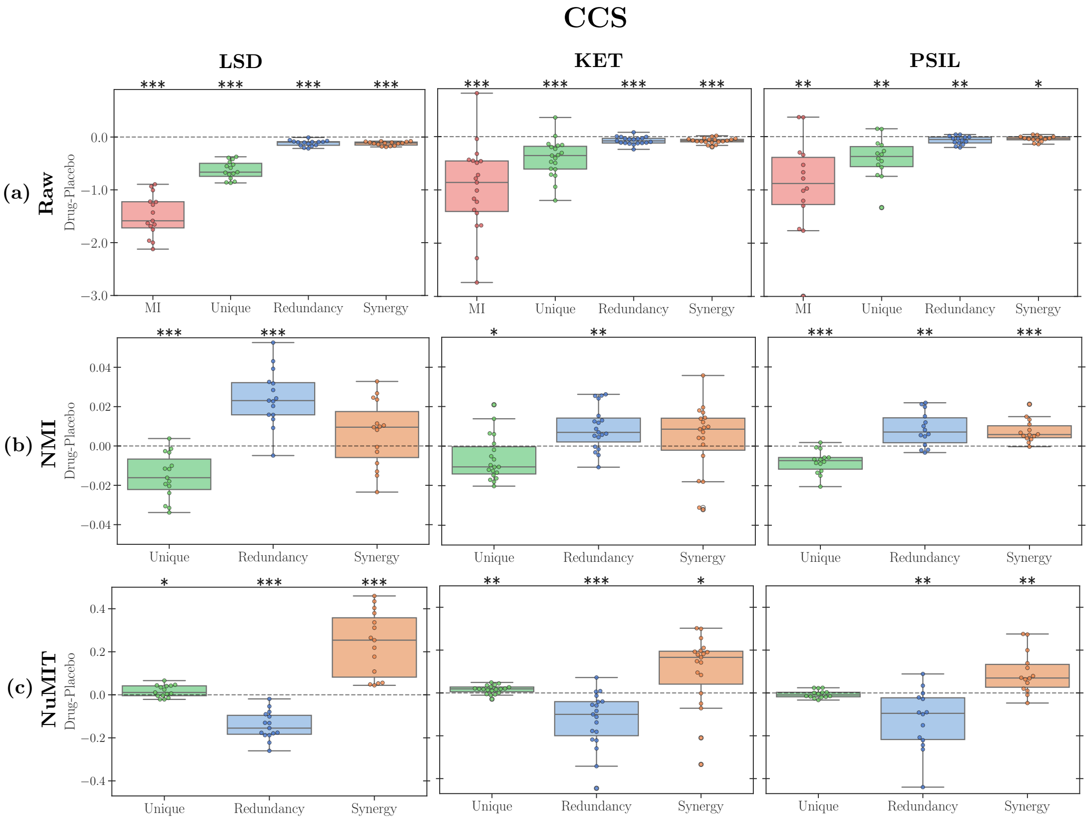

Fig B. PID atoms distribution for all subjects under different drugs and placebo effects using the CCS PID. From left to right, results refer to LSD, ketamine, and psilocybin drugs. Panel rows represent (a) the raw values of PID atoms, (b) the NMI-normalised PID atoms, and (c) the NuMIT-normalised PID atoms. The dashed black lines are drawn at zero. (P-values calculated with a one-sample t-test against the zero-mean null hypothesis. *: $p < 0.05$; **: $p < 0.01$; ***: $p < 0.001$).

Regression model for DEP−MMI and CCS – DEP

Here we present the comparisons between DEP vs. MMI and CCS vs. DEP PID definitions with the LME and the OLS regression model introduced in Sec. *Case study: Information decomposition in cortical dynamics*, with appropriate changes to the atoms in Eqs. (12)-(28). Since redundancies and unique information follow different definitions and interpretations in these formulations, we only focus on the comparisons between synergy atoms, fitting the LME, performing the regression, and presenting the results in Fig D.

Overall, LME models indicate that the interaction role between normalisation and PID definition play again a fundamental role in the model, displaying a strong statistical significance. Looking at the different drugs separately, we see that in both DEP-MMI and CCS-DEP cases NuMIT performs better than NMI, except for psilocybin in the DEP-MMI case (top right of Fig Da), in which both normalisations behave very similarly. Analogously to the results in the main text, we present in Tables A and B the slopes of the regression lines, i.e. the Pearson coefficient of the PID atoms, as well as the p-value of the interaction coefficients.

These results, along with the ones obtained in Sec. *Case study: Information decomposition in cortical dynamics*, support the idea that the null model normalisation

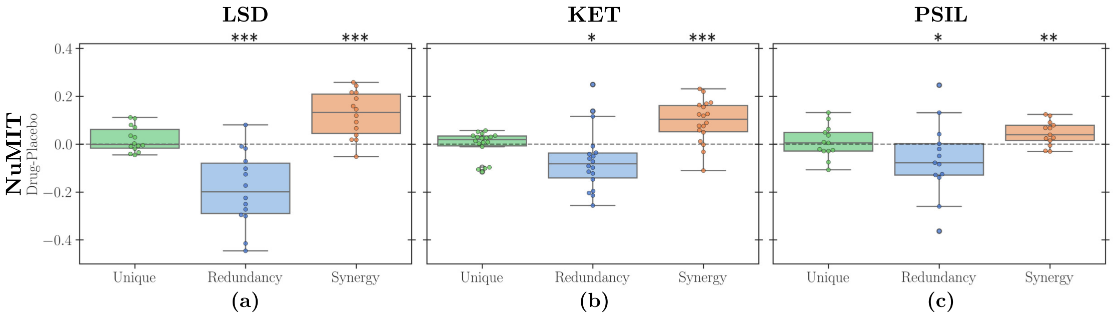

Fig C. Data-driven NuMIT-normalised PID atoms distribution for all subjects under different drugs and placebo effects using the MMI PID. Here, the null distributions were constructed using the evolution coefficients A of the real systems. Results refer to (a) LSD, (b) ketamine, and (c) psilocybin drugs. The dashed black lines are drawn at zero. (P-values calculated with a one-sample t-test against the zero-mean null hypothesis. *: $p < 0.05$; **: $p < 0.01$; ***: $p < 0.001$).

might be able to capture the emergent components of the system more deeply, as it yields synergy atoms that more consistent across different PID definitions.

PID atom	Drug	Correlations		p-value
		NMI	NuMIT	
Synergy	ALL	-0.115	0.113	0.001
	LSD	0.032	0.638	0.10
	KET	0.502	0.689	0.50
	PSIL	0.631	0.575	0.87

Table A. Pearson correlations and p-values of the interaction terms between Normalisation and PID atom for the LME (ALL) of Eq. (12) and for regression model (LSD, KET, PSIL) of Eq. (28) (replacing Δ_{CCS} with Δ_{DEP}) for synergy (DEP-MMI comparison).

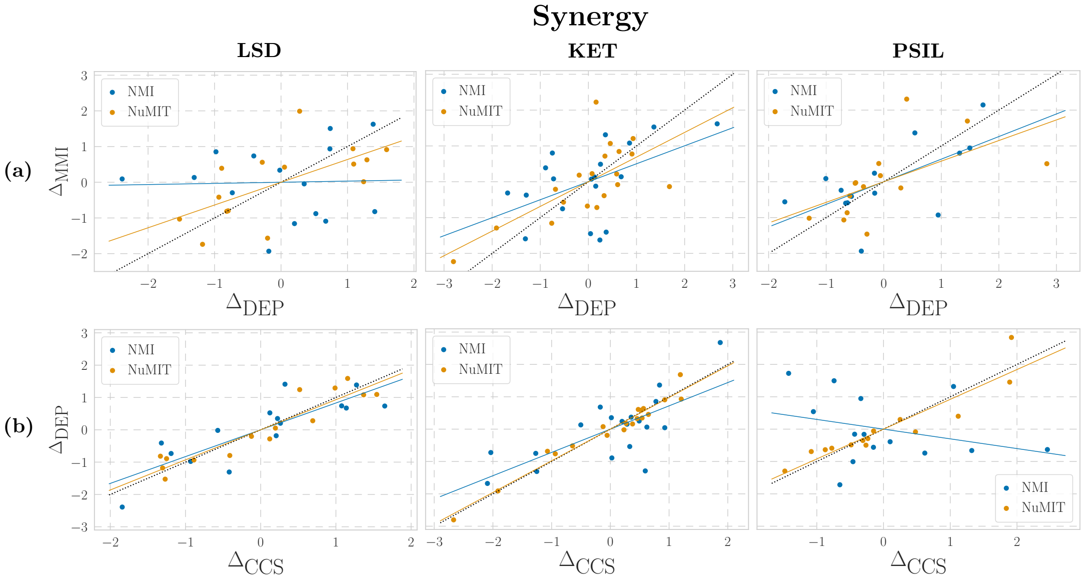

Fig D. Regression models for NMI- and NuMIT-normalised synergies between (a) DEP and MMI (b) CCS and DEP PID definitions, for LSD, ketamine, and psilocybin. Δ denotes the differences between drug and placebo in PID atoms obtained with any PID (MMI, CCS, or DEP). The black dotted line is the bisector of the first quadrant.

PID atom	Drug	Correlations		p-value
		NMI	NuMIT	
Synergy	ALL	-0.091	0.290	0.001
	LSD	0.830	0.931	0.59
	KET	0.720	0.978	0.15
	PSIL	-0.300	0.921	0.004

Table B. Pearson correlations and p-values of the interaction terms between Normalisation and PID atom for the LME (ALL) of Eq. (12) and for regression model (LSD, KET, PSIL) of Eq. (28) (replacing Δ_{MMI} with Δ_{DEP}) for synergy (CCS-DEP comparison).

B Further validations on synthetic systems

To further show the superiority of NuMIT over NMI, we demonstrate its operation on three basic Gaussian systems, each designed to exhibit predominantly redundant, unique, or synergistic interactions. The aim is to both inspect the quantile of the predominant PID atom with respect to the null distribution, as well as test whether our model can capture the information structure of the system independently of the noise level. Results are then compared with NMI-normalised quantities.

As a first test, we expect redundancy to be highest when the two sources are highly correlated and are mixed via the same coefficients in A, thus providing the same information about T. For this purpose, we can choose:

$$A = \begin{pmatrix} 0.49 & 0.49 \end{pmatrix} \; \Sigma_S = \begin{pmatrix} 1 & 1-\delta \\ 1-\delta & 1 \end{pmatrix} \; \Sigma_\epsilon = 0.1 \,, \tag{A}$$

where δ is set to 0.15 to allow a small variability of unique and synergy atoms. We then

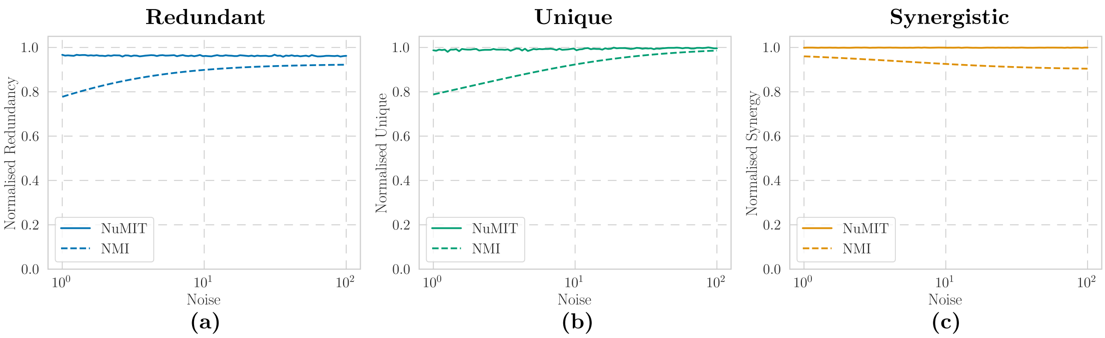

Fig E. Quantiles of the PID atoms for Gaussian models of Eq. (6) for various noise levels $g \in [1, 100]$. (a) Predominantly redundant system (Eq. (A)), (b) predominantly unique system (Eq. (B)), (c) predominantly synergistic system (Eq. (C)).

calculate PID atoms using the null-model procedure for a range of values of $g \in [1, 100]$, and compare it with NMI results. Fig Ea shows that normalised redundancy is approximately 1, while NMI redundancy varies with the noise level.

Next, a predominantly unique system is one where a source is responsible for the majority of the total mutual information, while the other is uncorrelated with the target. We can achieve this by setting

$$A = \begin{pmatrix} 0.01 & 0.9 \end{pmatrix} \quad \Sigma_S = \begin{pmatrix} 1 & 0 \\ 0 & 1 \end{pmatrix} \quad \Sigma_\epsilon = 10^{-4}\,. \tag{B}$$

We perform the same procedure as before and obtain Fig Eb, where the unique line is only the unique information brought by Y. Again, we observe that the normalised unique information is close to 1 and independent of the noise, while NMI normalisation provides a noise-dependent quantity.

Finally, we focus on a predominantly synergistic system. This can be obtained by highly anticorrelating the sources together while symmetrically correlating them with the target through A:

$$A = \begin{pmatrix} 0.49 & 0.49 \end{pmatrix} \quad \Sigma_S = \begin{pmatrix} 1 & -0.8 \\ -0.8 & 1 \end{pmatrix} \quad \Sigma_\epsilon = 0.01\,, \tag{C}$$

Fig Ec reports the results of the NuMIT normalisation, confirming that the system is indeed maximally synergistic among the null models. At the same time, NMI synergy decreases for larger values of noise.

Hence, as well as producing the expected values, in all three cases above the normalised PID atoms indicate that the effects of the noise and mutual information on the decomposition are suppressed, satisfying the criteria outlined in the main article.

C Further sweep analyses

Gaussian distributions

In this section, we present further analyses of our proposed normalisation method on the Gaussian system of Eq. (3). In particular, verifying the independence of the normalised atoms of the noise of the system for an additional case of MMI, as well as using DEP and CCS definitions.

We begin by studying the MMI PID. As the system considered in Eq. (4) of Sec. *A simple example* is highly symmetric and might be hiding non-trivial dependencies between the sources, we perform the same noise sweep procedure shown in Fig. 1 with the following randomly sampled parameters

$$A = \begin{pmatrix} -0.3 & 0.9 \end{pmatrix} \quad \Sigma_S = 50 \begin{pmatrix} 5 & 7 \\ 7 & 11 \end{pmatrix}, \tag{D}$$

Examining the results shown in Fig F, we observe again that the null-normalised atoms assume a constant value for different values of noise, whereas the NMI ones vary.

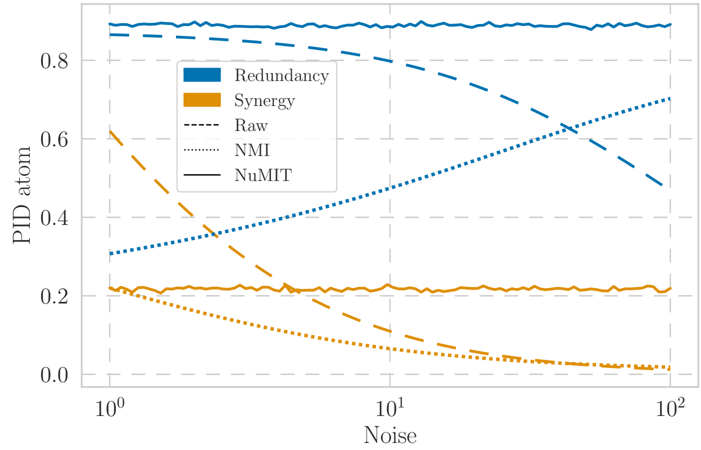

Fig F. Raw, NMI and Null-model redundancy and synergy atoms calculated for an asymmetric system given by Eqs. (3) and (D) for noise level $g \in [1, 100]$ and MMI definition.

Turning to other PID definitions, we now test our model performing the same procedure with DEP and CCS formulations. Using the parameters of Eq. (4), we obtain the results reported in Fig G. We observe that for CCS, synergy remains nearly constant, while redundancy exhibits gradual growth. Nonetheless, its increase is significantly smaller compared to that of the NMI redundancy.

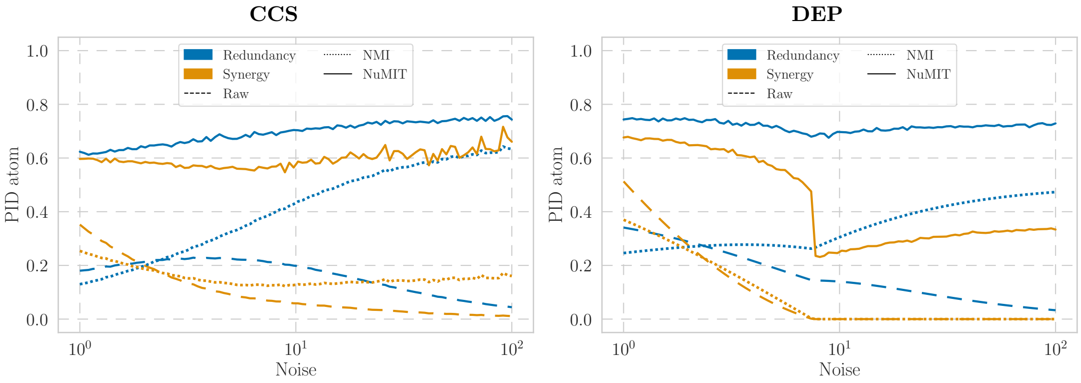

(a) **(b)**

Fig G. Raw, NMI and Null-model redundancy and synergy atoms calculated for the symmetric system given by Eqs. (3) and (4) for noise level $g \in [1, 100]$ and (a) CCS and (b) DEP definitions.

On the other hand, in the DEP case, although redundancy shows near-flat behaviour, the null-normalised synergy presents a sudden drop when its raw value becomes zero. Although this is not the desired behaviour, it is not surprising that it happens when the PID component vanishes: as our model is based on the assessment of percentiles, the calculations are sensitive to small variations in the distributions, particularly when numerical values of the atoms are of small magnitude, which may result in unstable estimates. Moreover, we underline that DEP formulation entails a minimisation procedure over the edges of a constraint lattice, which can indeed lead to discontinuous jumps when the TMI of the system changes.

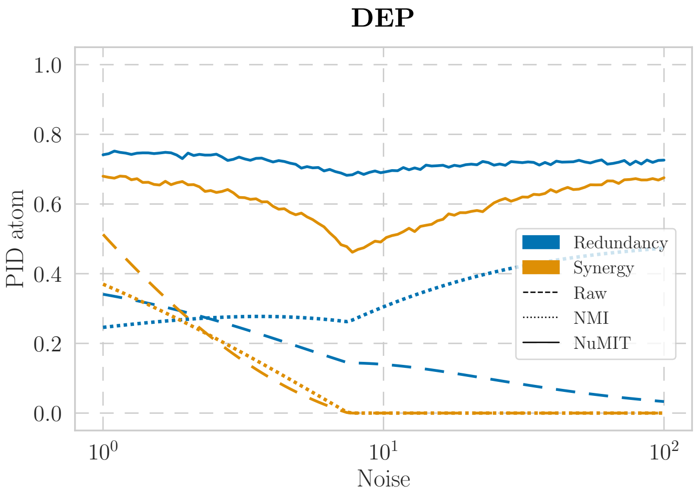

Fig H. Identical to Fig Gb, but calculated by setting the quantile of repeated values to its upper bound. The sharp drop in the NuMIT-synergy is drastically reduced (c.f. Fig Gb).

We also underline that part of this behaviour arises from the way quantiles are defined when repeated values occur: the estimate is placed at the midpoint of the block of identical values, and, since many null systems exhibit vanishing atoms, the transition of an atom from being slightly above zero (and therefore ranked above the ensemble of zeros) to being zero produces an abrupt jump in the quantile. This issue can be alleviated by defining the quantile as the upper bound of the repeated-value range rather than its midpoint (Fig H).

Whether the quantile is set to the mean or the maximum of the repeat values can be decided on a case-by-case basis, depending on the behaviour of the raw atoms. Throughout this work, we favoured the former approach to avoid overestimating the contributions of each atom. However, while this choice can influence the absolute values of the normalised atoms, it does not affect relative comparisons across different systems. Finally, we note that this behaviour is a special scenario, as zero is the only accumulation point of the atomic distributions, and in the vast majority of cases, atoms do not vanish by varying the noise level. Specifically, we remark that this investigation only concerns synthetic systems and does not affect the neural results obtained, since none of the atoms in real systems are exactly zero.

In sum, caution is recommended when a PID atom value becomes zero. From a practical point of view, it might be more meaningful to study these cases separately without the need for a normalisation technique.

VAR processes

In Fig. 2c-2d in the main article, we showed how PID and NMI-normalised PID atoms change on average for various values of TMI. For completeness, here we report a similar result for the VAR models (Fig I).

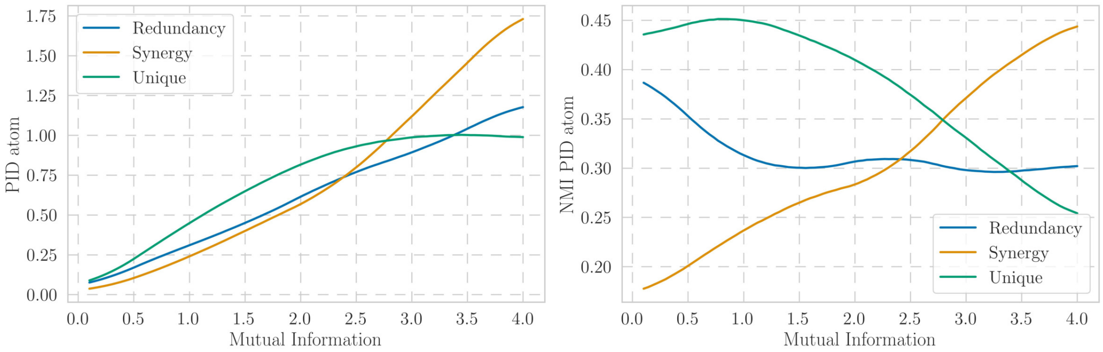

(a) **(b)**

Fig I. (a) PID-atom averages for different values of mutual information over random VAR systems of dimension $d = 2$. (b) Same as (a) but with NMI-normalised PID atoms.

Similarly to the Gaussian scenario, for higher TMI synergy tends to dominate the information of the system, while redundancy and unique information approach a constant value. On the other hand, systems with small TMI are predominantly redundant and unique.

D Higher-dimensional systems

Except for the VAR model results on neural data, so far the operation of the null model has been shown for univariate systems. In this section we briefly show that our procedure can be easily generalised to multivariate sources, producing the desired results.

As information-theoretic quantities depend on the dimensionality of the random variables involved, we expect the shapes of the PID-atom distributions shown in Fig. 2c to change with the dimensionality of the sources (note that in all cases we perform a two-source PID, with multivariate sources). Considering the Gaussian system of Eq. (6), we repeat the same procedure followed in Sec. *On the distribution of PID atoms* and analyse the average value of the null distributions for various dimensions d_S of the multivariate sources. The results reported in Fig J depict a clear trend: synergy and redundancy increase for larger systems, while unique information becomes smaller. Moreover, one can notice that the non-linearity of their behaviour is accentuated by a larger system size.

Turning now to the null model implementation, we test the robustness of our method in the multivariate case by performing the usual noise sweep procedure on systems of size $d_S = 8$ and $d_S = 20$ (Fig K). Once again, the atoms normalised with the null model are approximately independent of the noise strength, while raw and NMI-normalised values vary greatly.

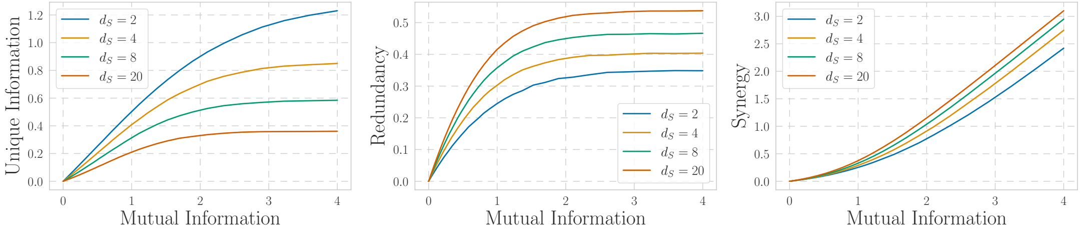

(a) (b) (c)

Fig J. Averages of PID atoms for different values of mutual information compared across Gaussian systems of various sizes for (a) redundancy, (b) unique information, (c) synergy.

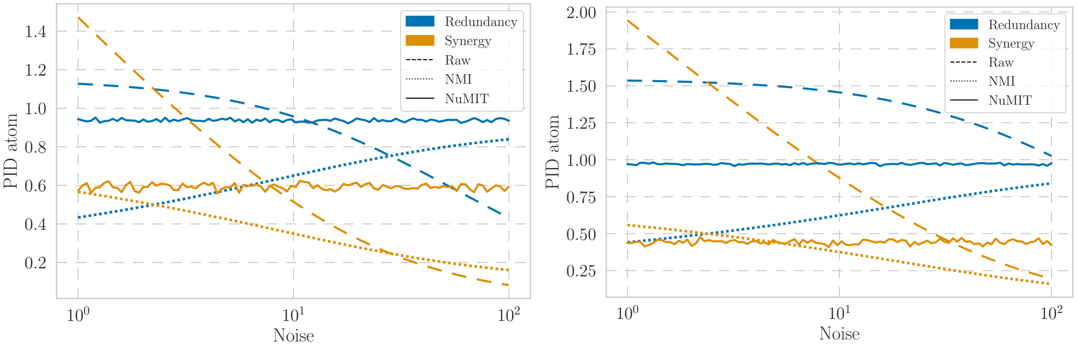

(a) (b)

Fig K. Raw, NMI and Null-model redundancy and synergy atoms calculated for the system given by Eq. (6) for noise level $g \in [1, 100]$ and (a) $d_S = 8$ and (b) $d_S = 20$. Parameters $A, \Sigma_S, \Sigma_\epsilon$ were randomly sampled.

E NuMIT robustness to null parameters

Throughout the whole manuscript, for Gaussian systems we focused on null models generated by sampling Gaussian coefficients A^{ij} and a noise term with a specific structure. A natural question would be whether these choices affect the outcome of the normalisation procedure, and what would happen if different parameters and distributions are employed.

Hence, here we reproduce the analysis performed on the system of Eq. (4) with null models generated differently. As outlined in Sec. *The Gaussian case*, the null coefficients $\tilde{A}^{ij}$ were randomly sampled from a mean-zero unit-variance normal distribution $\mathcal{N}(0,1)$, and the noise covariance $\tilde{\Sigma}_\epsilon$ from a Wishart distribution $W_{d_T}(\mathbb{1}_{d_T \times d_T}, d_T)$ with the identity $\mathbb{1}_{d_T \times d_T}$ as the base matrix. We now relax these constraints and consider the following scenarios:

1. $\tilde{A}^{ij} \sim \mathcal{N}(0,\sigma)$ and $\tilde{\Sigma}_{\boldsymbol{\epsilon}} \sim W_{d_T}(\mathbb{1}_{d_T \times d_T}, d_T)$;
2. $\tilde{A}^{ij} \sim \mathcal{N}(0,\sigma)$ and $\tilde{\Sigma}_{\boldsymbol{\epsilon}} \sim W_{d_T}(\mathcal{W}, d_T)$, with $\mathcal{W} \sim W_{d_T}(\mathbb{1}_{d_T \times d_T}, d_T)$;
3. $\tilde{A}^{ij} \sim \mathcal{U}(-a,a)$ and $\tilde{\Sigma}_{\boldsymbol{\epsilon}} \sim W_{d_T}(\mathbb{1}_{d_T \times d_T}, d_T)$;
4. $\tilde{A}^{ij} \sim \mathcal{U}(-a,a)$ and $\tilde{\Sigma}_{\boldsymbol{\epsilon}} \sim W_{d_T}(\mathcal{W}, d_T)$, with $\mathcal{W} \sim W_{d_T}(\mathbb{1}_{d_T \times d_T}, d_T)$;

where we indicated with $\mathcal{U}(-a,a)$ the uniform distribution with support on the interval $[-a,a]$.

Following these rules, we can construct the various null distributions and normalise the PID atoms of Fig. 1. Additionally, for each of these cases, we also vary the parameters $a, \sigma \in (0, 10)$ to examine their effect. Results suggest that when the same type of distribution is used, i.e. Gaussian (1,2) or uniform (3,4), the obtained quantiles are highly consistent across a, σ, and the choice of the Wishart's base (Fig L). However, comparing the normal and uniform case, while the redundancy quantile remains the same, the one for synergy slightly changes.

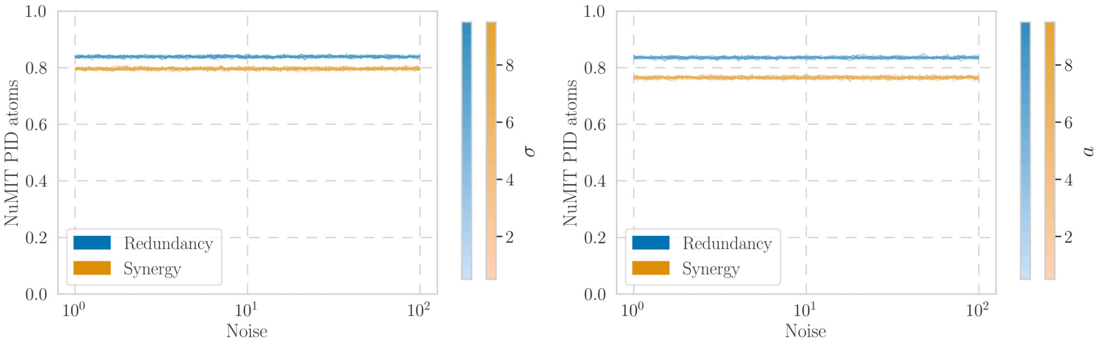

(a) **(b)**

Fig L. NuMIT PID atoms of the system of Eq. (4) computed from null models generated with: (a) Gaussian parameters with $\sigma \in (0, 10)$, and (b) uniform parameters with $a \in (0, 10)$. Both cases comprise noise covariances sampled from Wishart distributions with identity and random matrix as base matrix.

This should not come as a surprise, as every null model will show some dependency on the parameters chosen for their generation [3]. Moreover, we argue that this dependency does not pose a problem in empirical applications of the model. Null-normalised values are not intended to carry significance in absolute terms; rather, they serve as relative measures that enable comparisons across different systems, provided null models with same structural qualities are used. Along these lines, if we now consider the system of Eq. (D) and perform the different null normalisations as above, we notice that although the value of synergy slightly changes in this case too, the difference between the two systems' normalised quantities is the same both in the Gaussian and uniform case (Fig M).

Therefore, we showed that NuMIT provides a parameter-agnostic procedure to normalise PID atoms.

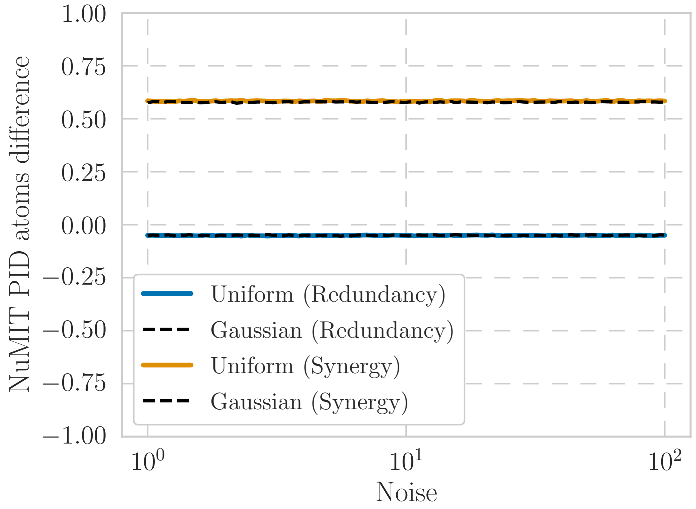

Fig M. Differences of the NuMIT PID atoms of the systems of Eqs. (4)- (D) computed from null models generated with Gaussian and uniform parameters.

F Null-model normalisation for discrete random variables

Finally, we suggest a possible implementation of our technique for discrete random variables. We first present the mathematical formulation (Sec. *Normalisation for discrete variables*), then validate the model on synthetic systems in Sec. *Validation in synthetic systems.*

Normalisation for discrete variables Let X and Y be two discrete variables jointly sampled from a multinomial distribution $\text{Mult}(n, k, p_1, ...p_k)$, where n is the number of trials, k the number of possible events, and $p_1, ...p_k$ the probabilities associated to each event, and take f a Boolean function of X and Y, e.g. a two-input logic gate. We define the target T as

$$T = Z + \epsilon\,, \tag{E}$$

with $Z = f(X;Y)$, and where ϵ is a discrete noise term that flips the value of Z with probability p_ϵ. The probabilities $p_1, ..., p_k$ that govern the statistics of X, Y can be sampled from a Dirichlet distribution $\text{Dir}(\alpha_1, ..., \alpha_k)$ defined on a $k-1$ simplex. For simplicity, we take $\alpha_i = \alpha\ \forall i = 1, ..., k$. The entropies can be computed using the definition in Eq. (13):

$$\begin{aligned} H(T) = &- p(T=0)\log p(T=0) + \\ &- p(T=1)\log p(T=1)\,, \end{aligned} \tag{F}$$

$$H(T|S) = - p_\epsilon \log p_\epsilon - (1-p_\epsilon)\log(1-p_\epsilon)\,, \tag{G}$$

where $p(T=0) = p(Z=0)(1-p_\epsilon) + p(Z=1)p_\epsilon$, and analogous for $p(T=1)$. Once the function f is chosen, mutual information can be calculated as the difference of Eqs. (F) and (G) (see Eq. (14) in Sec. *Materials and Methods*).

The construction of the null model is then analogous to the cases already described: sampling probabilities $p_1, ..., p_k$ from $\text{Dir}(\alpha)$ and choosing a random gate f, we treat p_ϵ as the parameter used for the optimisation, tuning it to produce the desired value of MI.

X	Y	Z_1	Z_2	Z_3	Z_4	Z_5	Z_6	Z_7
0	0	0	0	0	0	1	1	1
0	1	1	0	1	1	0	1	1
1	0	1	1	0	1	1	0	1
1	1	0	1	1	1	1	1	0

Table C. 2-input logic gates used for the null model.

Finally, marginal mutual information $I(X;T)$, $I(Y;T)$ can be computed from the definition of entropy, and PID is performed. In the following section, we provide a practical example of the procedure.

Validation in synthetic systems We illustrate here the application of the null model normalisation on discrete systems of the form given by Eq. (E), in which f is a 2-input logic gate.

Logic gates are a class of discrete systems that perform Boolean operations on binary inputs, being often used to study the behaviour of information-theoretic quantities in simple cases [2]. In our perspective, we consider binary inputs X and Y, whose probability is sampled from a Dirichlet distribution, employing 2-input logic gates as proxies to test the functioning of the null model. Analogously to the Gaussian system in Sec. *Validation on synthetic systems*, we focus on maximal synergistic, redundant, and unique information systems, performing the null model procedure and expecting an atom quantile close to the unit value.

Since the number of logic gates for binary variables is relatively small ($2^4 = 16$), we construct the null model considering all of such systems. However, of those 16, we can neglect both the tautology and contradiction, as the output is independent of the input and therefore $I(T;X,Y) = 0$, and those gates which are the same under the symmetry $0 \leftrightarrow 1$, as it leaves the statistical structure of the model invariant. Hence, we are left with only 7 gates which are reported in Table C; among those, we can recognise the XOR (Z_1) and the OR (Z_4) gates.

In Sec. *Normalisation for discrete variables* we showed how the mutual information between source and targets can be computed. For the marginal mutual information $I(X;T)$ and $I(Y;T)$, we make use of

$$I(X;T) = H(T) - H(T|X)\,, \tag{H}$$

recalling the definition of entropy and conditional entropy Eqs. (13) and (15), and manually calculating their values using the gate definitions of Table C. After an analogous procedure is performed for $I(Y;T)$, the PID decomposition is applied.

Analogous to Sec. *Validation on synthetic systems*, we analyse whether the null normalisation is robust to noise changes. Starting with a predominantly redundant system, we take the table of truth given by Z_5, with probabilities $p_{00} = p_{11} = \delta/2$, $p_{01} = p_{10} = (1-\delta)/2$, where δ is a small positive number (0.01) and p_{ab} indicates the joint probability $p(X = a, Y = b)$. This way we obtain a system in which X and Y provide the same amount of information due to symmetry, with minimal synergistic component as $\{00\}$ and $\{11\}$ inputs are suppressed by the low probability. For each level of noise, we perform the decomposition and run the null model over the space of the 7 possible logic gates, obtaining a distribution of redundancy atoms and the quantiles shown in Fig. Na. As expected, we observe a high quantile (~ 1) for the maximally redundant system, independently of the noise level. Although synergy atom shows some fluctuations, redundancy is clearly greater than the other two atoms throughout.

Correspondingly, as a predominantly unique system we consider the Z_2 gate with equal probabilities $p_{00} = p_{01} = p_{10} = p_{11} = 0.25$. In fact, if not for the possible flip

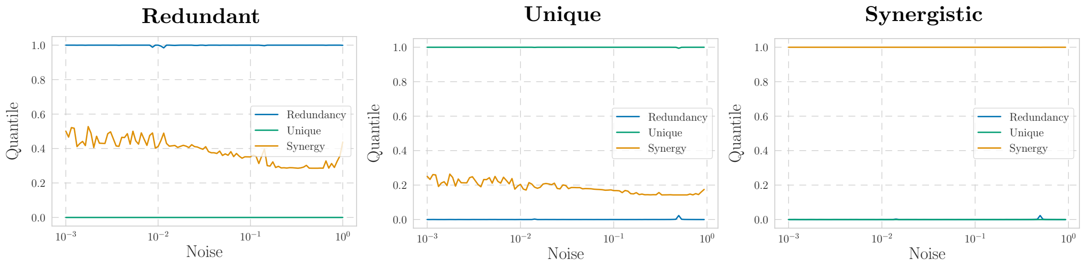

(a) (b) (c)

Fig N. Quantiles of the PID atoms for discrete models of Eq. (E) for various noise levels $p_\epsilon \in [0.001, 1)$. (a) Predominantly redundant system (Z_5), (b) predominantly unique system (Z_2), (c) predominantly synergistic system (Z_1).

induced by the noise term ϵ, the knowledge of X would completely determine the output T. Spanning $p_\epsilon \in [0.001, 1)$ and proceeding with the null model construction, we report the atoms' behaviour in Fig. Nb, verifying the expected trend.

At last, we turn to a purely synergistic system, i.e. a model in which the knowledge of one variable does not convey any information on the outcome of the target. This is obtained considering the XOR (Z_1) with independent probabilities $p_{00} = p_{01} = p_{10} = p_{11} = 0.25$. Following the same steps as above we obtain Fig. Nc, confirming the expected results.

References

1. James RG, Emenheiser J, Crutchfield JP. Unique information via dependency constraints. Journal of Physics A: Mathematical and Theoretical. 2018;52(1):014002.

2. Ince RA. Measuring multivariate redundant information with pointwise common change in surprisal. Entropy. 2017;19(7):318.

3. Rubinov M, Sporns O. Complex network measures of brain connectivity: uses and interpretations. Neuroimage. 2010;52(3):1059–1069.
